# Current utilization of interosseous access in pediatrics: a population-based analysis using an EHR database, TriNetX

**DOI:** 10.1186/s12245-022-00467-9

**Published:** 2022-11-29

**Authors:** Meloria Hoskins, Samantha Sefick, Adrian D. Zurca, Vonn Walter, Neal J. Thomas, Conrad Krawiec

**Affiliations:** 1 Penn State College of Medicine, 500 University Drive, P.O. Box 859, Hershey, PA USA; 2 Pediatric Critical Care Medicine, Department of Pediatrics, Penn State Hershey Children’s Hospital, 500 University Drive, P.O. Box 850, Hershey, PA USA; 3 Division of Biostatistics and Bioinformatics, Department of Public Health Sciences, Pennsylvania State University College of Medicine, 500 University Drive, Hershey, PA USA; 4 Department of Public Health Sciences, Pennsylvania State University College of Medicine, 500 University Drive, Hershey, PA USA

**Keywords:** Interosseous, Emergent access, Critical care, Pediatrics, Neonates, Complications

## Abstract

**Background:**

When central or peripheral intravenous access cannot be achieved in a timely manner, intraosseous (IO) access is recommended as a safe and equally effective alternative for pediatric resuscitation. IO usage and its complications in the pediatric population have been primarily studied in the setting of cardiac arrest. However, population-based studies identifying noncardiac indications and complications associated with different age groups are sparse.

**Results:**

This was a retrospective observational cohort study utilizing the TriNetX® electronic health record data. Thirty-seven hospitals were included in the data set with 1012 patients where an IO procedure code was reported in the emergency department or inpatient setting. The cohort was split into two groups, pediatric subjects < 1 year of age and those ≥ 1 year of age. A total incidence of IO line placement of 18 per 100,000 pediatric encounters was reported. Total mortality was 31.8%, with a higher rate of mortality seen in subjects < 1 year of age (39.2% vs 29.0%; *p* = 0.0028). A diagnosis of cardiac arrest was more frequent in subjects < 1 year of age (51.5% vs 38.0%; *p* = 0.002), and a diagnosis of convulsions was more frequent in those ≥ 1 of age (28.0% vs 13.8%; *p* <0.01). Overall, 29 (2.9%) subjects had at least one complication.

**Conclusions:**

More IOs were placed in subjects ≥ 1 year of age, and a higher rate of mortality was seen in subjects < 1 year of age. Lower frequencies of noncardiac diagnoses at the time of IO placement were found in both groups, highlighting IO may be underutilized in noncardiac settings such as convulsions, shock, and respiratory failure. Given the low rate of complications seen in both groups of our study, IO use should be considered early on for urgent vascular access, especially for children less than 1 year of age.

**Supplementary Information:**

The online version contains supplementary material available at 10.1186/s12245-022-00467-9.

## Background

Obtaining vascular access is often a vital first step to providing fluid resuscitation and medications for children presenting with a wide variety of emergent clinical conditions [1–5]. Peripheral venous access is the most common intravascular access used in the pediatric population. However, smaller veins, patient anxiety, changes in vascular tone, and particular physiological characteristics (i.e., increased subcutaneous adipose tissue thickness) make obtaining venous access difficult in pediatric emergencies [6, 7]. When central or peripheral intravenous access cannot be achieved in a timely manner, intraosseous (IO) access is recommended as a safe and equally effective alternative for delivering fluids and medications [8–12]. This includes high-risk populations experiencing cardiac arrest, respiratory failure, sepsis, shock, major traumatic injuries, and status epilepticus [6, 13].

The use of intraosseous space to administer fluids and medications was first described in the 1920s. It became more frequently utilized in North America in the 1940s when research demonstrated its safety in pediatric patients [14–16]. The IO route allows access to the systemic venous circulation via the placement of a hollow needle through the cortex of the bone into the medullary cavity, usually through the proximal tibia or distal femur. A network of intramedullary venous sinusoids drains directly into the central venous system, allowing blood sampling and the ability to administer any traditionally intravenous fluids or medications [7, 17, 18]. Because it allows quick and safe access, the most recent American Heart Association’s Pediatric Advanced Life Support (AHA PALS) update recommends that IO cannulation be the vascular access method of choice when intravenous access has not been established within 30 s of resuscitative care [11, 19]. Despite the benefits, IO access does not come without risk. Studies have reported the most common complications of IO usage include extravasation, infection (cellulitis and osteomyelitis), compartment syndrome, lower extremity fractures, and thrombosis [6, 7, 20–22].

Many of the current published studies mainly focus on IO usage in the setting of cardiac arrest, respiratory failure, and trauma [20–23]. While these conditions are the most widely known indications for IO access in children, its use in pediatrics has been expanded to include severe hemodynamic disorders, neurological compromise, shock, and severe bleeding [7, 12, 14, 17, 24]. The frequency of its use for noncardiac arrest indications, the type of patient population, and the complication rate are presently unknown. Examining the frequency, patterns of care, and complications of IO usage in cardiac and noncardiac settings while comparing different age groups may assist in understanding how IO usage has evolved in pediatric practice.

The objective of this present study is to utilize an electronic health record (EHR) database, TriNetX, and to evaluate the current usage, clinical characteristics, and complications of pediatric IO access in multiple healthcare organizations (HCOs). By stratifying the study population by age, we aim to better understand any differences in patterns of care and outcomes after IO use between age groups. We hypothesize that IO access is primarily used in younger pediatric patients, complications are rare, and the use of IO in this population has expanded beyond cardiac indications.

## Materials and methods

### Study design

This is a retrospective observational cohort study utilizing the TriNetX® electronic health record (EHR) data of pediatric subjects aged 0 to 18 years who were found to have an IO procedural code [Common Procedural Terminology (CPT): 36680]. TriNetX® is a global federated research network that provides EHR data elements (i.e., diagnoses, procedures, laboratory values) of approximately 68 million patients in 56 large healthcare organizations predominately in the USA. The data is de-identified and aggregated within a real-time user-friendly browser-based software accessible to the study authors. Because no protected health information is provided, we received a waiver from the Penn State Health Institutional Review Board (IRB) to perform this study.

### Data collection

TriNetX provided a de-identified dataset of electronic medical records (diagnoses, procedures, medications) from all eligible patients from 37 US HCOs up to the date of the database query. TriNetX is compliant with United States federal law which protects the privacy and security of healthcare data, the Health Insurance Portability and Accountability Act (HIPAA). TriNetX is certified to the ISO 27001:2013 standard and maintains an information security management system (ISMS) to ensure the protection of the healthcare data it has access to and to meet HIPAA Security Rule requirements. Any aggregate data displayed on the TriNetX platform, or any patient level data provided within the data set generated by the TriNetX platform, only contains de-identified data as per the de-identification standard defined in Section §164.514(a) of the HIPAA Privacy Rule. A formal determination by a qualified expert as defined in Section §164.514(b)(1) of the HIPAA Privacy Rule was performed and attested to the process by which the data is de-identified.

On August 12, 2021, we analyzed the following EHR data: age, sex, race, ethnicity, International Classification of Diseases (ICD) 9th and 10th edition diagnostic codes on the same day of the IO procedure and up to and including 3 months thereafter, non-IO critical care procedures required, medications, patient encounters reported, and mortality. Ages were provided in years. The data were de-identified, and no date of birth was provided; thus, ages were approximate for subjects older than 1 year of age. For example, a child born in 2018 with an IO procedure code is noted on January 1, 2021; the subject was determined to be 3 years of age. In children less than 1 year of age, we were unable to calculate the exact months; thus, they were given an age of 0. We split the cohort into two groups, pediatric subjects less than 1 year of age and those equal to or greater than 1 year of age. Due to the number of different codes that may be utilized for each clinical critical condition, complex chronic conditions, procedures, and medication, we categorized each code as outlined in Supplemental Table 1. Complex chronic conditions were defined based on a list developed by Feudtner et al., which incorporates any medical condition that is expected to last at least 12 months [25].

This dataset does not include IO use during operating room procedures because the TriNetX database does not include the timing of procedure code placement and does not report the operating room as a potential location. Because not all encounter types may be reported in this dataset (i.e., listed as unknown), we opted to include all subjects with an unknown and inpatient encounter type. This approach was undertaken as most subjects receive an IO in an emergency setting; however, if the coding/billing did not occur during the emergency setting, the encounter type was labeled unknown in the database.

To gain an understanding of the incidence of pediatric ED visits and admissions where interosseous access was utilized, on October 4, 2021, we used TriNetX browser-based real-time analytical features to determine the total number of ED visits and inpatient admissions within the TriNetX database. We set the query to evaluate this within the same time period of our initial analysis.

### Statistical analysis

Summary counts and percentages of demographic characteristics, diagnostic categories, and other variables of interest in the two age group categories were computed with RStudio version 1.4.1106 [26]. Fisher’s exact test was used to assess the statistical significance of bivariate associations. When assessing race only, subjects of “unknown” race were removed from the analysis. Because of the exploratory nature of this study, no adjustment for multiple testing was applied.

## Results

### Patient demographics

There were 37 hospitals in the data set with 1012 pediatric patients where an IO procedure code was reported in the emergency department or inpatient setting. Those less than 1 year of age accounted for 268 (26.5%) of the study population, while 744 (73.5%) pediatric subjects were more than 1 year of age. Encounter type (i.e., emergency vs inpatient) was listed for 606 of the patients within the data set. In patients where encounter type was recorded, 364 (60.1%) IO lines were placed during ED visits, while 242 (39.9%) procedures occurred during inpatient encounters. Within the TriNetX database, there were 4,114,773 pediatric ED visits, and 1,428,217 pediatric admissions, generating an IO line placement incidence of 9 per 100,000 ED visits and 17 per 100,000 pediatric inpatient admissions. There were 406 subjects that received IO where the encounter type was unknown. When including all pediatric subjects where an IO procedure code was reported, there is a total incidence of 18 per 100,000 pediatric encounters.

Table 1 presents demographics including gender, age, ethnicity, and race of the pediatric patients in this cohort. The mean age of the ≥ 1 year group was 3.9 years (± 4.3). While there were more IOs placed in subjects older than 1 year, the mortality rate was higher in patients less than 1 year of age (39.2% vs 29.0%; *p* = 0.0028).

**Table 1:** Clinical characteristics and demographics of pediatrics subjects with intraosseous access procedural code

	Total	< 1 year of age	≥ 1 year of age	*p*-Value
**Total number of subjects (*n*)**	1012	268	744	-
Less than 1 year of age	268	268 (100.0%)	0 (0.0%)	
1 to 5 years of age	584	0 (0.0%)	584 (78.5%)	
6 to 10 years of age	80	0 (0.0%)	80 (10.8%)	
11 to 15 years of age	55	0 (0.0%)	55 (7.4%)	
Above 16 years of age	25	0 (0.0%)	25 (3.4%)	
**Gender (*n*** **(%))**				0.423
Male	576 (56.9%)	157 (58.6%)	419 (56.3%)	
Female	434 (42.9%)	110 (41.0%)	324 (43.5)	
Unknown	2 (0.2%)	1 (0.4%)	1 (0.1%)	
**Mean age (mean +/− SD)**	2.8 ± 4.0	-	3.9 ± 4.3	-
**Ethnicity (*n*** **(%))**				0.0125
Hispanic or Latino	133 (13.1%)	23 (8.6%)	110 (14.8%)	
Not Hispanic or Latino	642 (63.4%)	171 (63.8%)	471 (63.3%)	
Unknown	237 (23.4%	74 (27.6%)	163 (21.9%)	
**Race (*n*** **(%))**				0.299
Black or African American	358 (35.4%)	104 (38.8%)	254 (34.1%)	
Other^b^	27 (2.7%)	7 (2.6%)	20 (2.7%)	
Unknown	193 (19.1%)	55 (20.5%)	138 (18.5%)	
White	434 (42.9%)	102 (38.1%)	332 (44.6%)	
**Diagnostic categories (*n*** **(%))**				
Diseases of the circulatory system	555 (54.8%)	174 (64.9%)	381 (51.2%)	0.000109
Diseases of the respiratory system	479 (47.3%)	120 (44.8%)	359 (48.3%)	0.354
Diseases of the nervous system	307 (30.3%)	55 (20.5%)	252 (33.9%)	< 0.01
Injury, poisoning, and certain other consequences of external causes	291 (28.8%)	52 (19.4%)	239 (32.1%)	< 0.01
Certain infectious and parasitic diseases	230 (22.7%)	54 (20.1%)	176 (23.7%)	0.269
**Encounter type** (*n* = 606^a^); **(*n*** **(%))**				
Emergency	364 (60.1%)	107 (39.9%)	257 (34.5%)	0.12
Inpatient	242 (39.9%)	63 (23.5%)	179 (24.1%	0.933
**Critical care services provided (*n*** **(%))**				
Critical care services	706 (69.8)	183 (68.3%)	523 (70.3%)	0.536
Invasive mechanical ventilation	550 (54.3%)	171 (63.8%)	379 (50.9%)	0.00034
Cardiopulmonary resuscitation	374 (37.0%)	123 (45.9%)	251 (33.7%)	0.000512
**Complex chronic diseases (*n*** **(%))**	310 (30.6)	75 (28.0%)	235 (31.6%)	0.28
**Deaths (*n*** **(%))**	321 (31.7%)	105 (39.2%)	216 (29.0%)	0.0028
Cardiac arrest	258 (25.5%)	91 (34.0%)	167 (22.4%)	-
Convulsions	65 (6.4%)	18 (6.7%)	47 (6.3%)	-
Respiratory failure	39 (3.9%)	7 (2.6%)	32 (4.3%)	-
Shock	30 (3.0%)	5 (1.9%)	25 (3.4%)	-

^a^Only 606 subjects were reported to have an emergency or inpatient encounter listed within the dataset. ^b^American Indian or Alaska Native, Asian, Native Hawaiian, or Other Pacific Islander

### Reported diagnostic codes

When grouped into diagnostic categories, diseases of the circulatory system and diseases of the respiratory system were the most common for both groups (Table 1). For those with a disease of the circulatory system, there was a higher frequency of subjects less than 1 year of age with an IO procedural code in comparison with those older than 1 year of age (64.9% vs 51.2%; *p* = 0.001). Conversely, diseases of the nervous system and injury, poisoning, and certain other consequences of external causes were both more frequent in subjects more than 1 year of age with an IO procedural code (33.9% vs 20.5%; *p* < 0.01 and 32.1% vs 19.4%; *p* < 0.01, respectively).

**Fig. 1:**
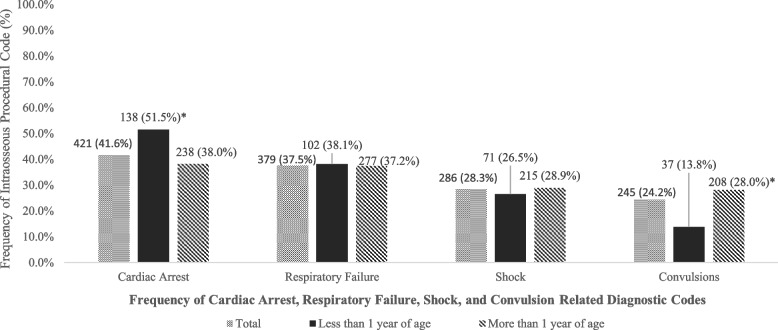
Frequency of cardiac arrest, respiratory failure, shock, and convulsion-related diagnostic codes on the same day of reported intraosseous access procedural code, *p < 0.05

The most common diagnosis at the time of IO placement for both groups was cardiac arrest, followed by respiratory failure, shock, and convulsions (Fig. 1). Of the intraosseous access procedure codes reported within the TriNetX database, a diagnosis of cardiac arrest was more frequent in children less than 1 year of age compared to children 1 year or older (51.5% vs 38.0%; *p* = 0.002). Additionally, a higher frequency of IO placement was identified in subjects more than 1 year of age with convulsions (28.0% vs 13.8%; *p* < 0.01).

### Reported medical services

Critical care procedure codes that were reported to be provided included critical care services, invasive mechanical ventilation, and cardiopulmonary resuscitation (Table 1). Both invasive mechanical ventilation and cardiopulmonary resuscitation were more commonly used in subjects less than 1 year of age (63.8% vs 50.9%; *p* = 0.00034 and 45.9% vs 33.7%; *p* = 0.000512, respectively).

### Reported medications administered

The most common type of medication reported to be administered on the same day IO access procedural code was placed was intravenous fluids for both groups, followed by cardiac arrest medications and electrolyte administration (Table 2). For subjects more than 1 year of age, anti-epileptics were used more frequently than in those less than 1 year of age (23.1% vs 17.5%; *p* = 0.0566).

**Table 2:** Type of medications administered on the same day intraosseous access procedural code placed

	< 1 year of age	≥ 1 year of age	*p*-value
Fluids	123 (45.9%)	346 (46.5%)	0.887
Cardiac arrest medications	115 (42.9%)	310 (41.7%)	0.773
Electrolyte administration	106 (39.6)	287 (38.6%)	0.826
Emergency anti-infectives	67 (25.0%)	212 (28.5%)	0.3
Anti-epileptics	47 (17.5%)	172 (23.1%)	0.0576
Cardiac therapy	31 (11.6%)	104 (14.0%)	0.347

### Intraosseous access diagnostic code complications

**Fig. 2:**
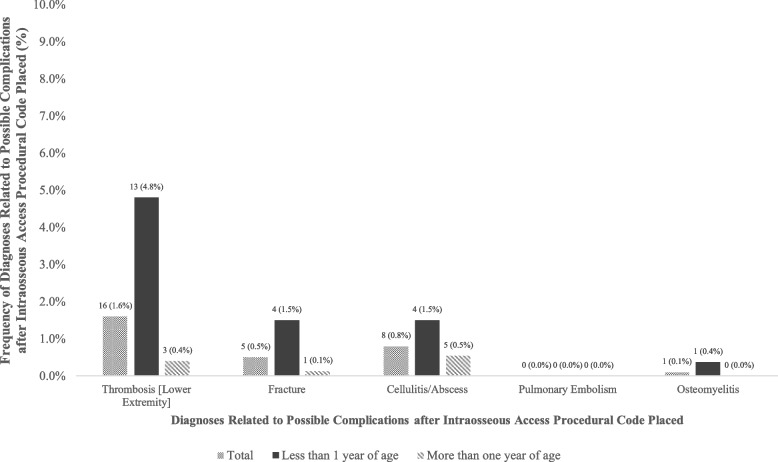
Frequency of diagnoses related to possible complications 1 day to 3 months after intraosseous access procedural code placed

Overall, 29 (2.9%) subjects had at least one complication on the same day of the IO procedure and up to and including 3 months thereafter. Of these, 21 (72.4%) subjects were less than 1 year of age, and 8 (27.6%) were greater than 1 year of age. Although the overall complication rate was rare in this cohort for both groups, potential IO complications reported include lower extremity thrombosis, fractures, cellulitis/abscess, pulmonary embolism, and osteomyelitis (Fig. 2).

## Discussion

Intraosseous access is often necessary to provide rapid resuscitation during pediatric emergencies. This study aimed to evaluate the current utilization, clinical characteristics, and complications of IO access in children among a large sample of hospitals using an EHR database, TriNetX. We found that more IO lines were placed in pediatric subjects ≥ 1 year of age, and a higher mortality rate was seen in subjects < 1 year. Additionally, our findings show a higher frequency of IO placement in subjects < 1 year of age with cardiac arrest and a higher frequency of IO placement in patients ≥ 1 year of age with convulsions.

By evaluating two different age groups within the pediatric population in a multicenter fashion, while restricted to the limitations of this dataset, we have expanded our knowledge of IO usage and outcomes. Furthermore, due to physiological reasons and the different approaches that are taken in emergency settings, it may be more reasonable to examine younger children separately from older children whenever possible. For example, it is more difficult to gain emergent vascular access in smaller and younger children due to smaller veins and involuntary movements. Thus, more IOs are placed in older subjects, and a higher rate of complications and deaths are observed in younger subjects, possibly due to the inability to achieve prompt access. By stratifying our population by age, we demonstrated differences in outcomes between these two groups, which is novel to this study.

While IO access is reported as a safe and rapid approach to pediatric resuscitation, there is minimal population-based data at present showing its utilization outside of cardiac arrest. Based on the lower frequencies of noncardiac diagnoses found in the study subjects at the time of IO placement, IO access may be underutilized in noncardiac settings such as convulsions, shock, and respiratory failure. Given the current AHA PALS guidelines and its interchangeable efficacy profile with other forms of vascular access, this should not be the case [9, 11, 19].

In the setting of neurological disease, a higher frequency of IO placement was seen in older pediatric subjects than in those less than 1 year of age in this study. There are many possible reasons for these findings. For example, administering intramuscular (IM) medications for seizure control is generally effective, but older children may need larger doses of medications, which may be easier to achieve with an IO. The lower frequency seen in subjects less than 1 year of age may highlight the difficulty in obtaining IO access in younger children, especially during excess involuntary muscle contraction. It is also possible there was a reluctance or discomfort in obtaining IO access in a young child. Alternatively, IO access may not have been considered necessary as intravenous access may have been successfully obtained, especially if the patient is otherwise healthy. Nevertheless, quick vascular access is essential to administer medications necessary to treat convulsions and prevent further complications, regardless of age. Thus, IO placement should be considered as soon as possible if other vascular access attempts appear to be futile when children experience convulsions, especially given the relatively low risk of complications in our study.

Despite including diagnoses other than cardiac arrest and respiratory failure in this study, the mortality rate was similar to other studies evaluating IO usage [23, 24]. Given the American Heart Association’s PALS recommendation, the mortality rate recorded in our study may seem high based on the similar safety profiles and efficacy of IO access compared to other intravascular access forms. For example, in the pediatric intensive care unit (PICU), the overall mortality rate has historically been reported to be 2% [11, 27]. However, children who present to the emergency department and inpatient setting often have preexisting medical conditions, comorbidities, and often are extremely ill (even before they reach the PICU). Vascular access in these patients can be challenging to achieve. Thus, the high mortality rate seen in both groups in this study population is likely a marker of the inherently poor prognosis of critically ill and medically complex pediatric patients who require IO access.

We identified possible complications 1 day to 3 months after IO use in this population, including lower extremity thrombosis, fracture, cellulitis/abscess, pulmonary embolism, and osteomyelitis. Our study demonstrates similar complication rates as previously reported [17, 18, 23, 28]. In this data set, the low incidence of complications reported may be explained by the mortality rate. More complications likely occurred with patients who did not survive and thus were not reported within the EHR. It should also be considered that not all complications were recorded, and perhaps the reported complication rate is lower than the actual rate. Finally, it is possible that the reported complications were unrelated to IO placement. Nonetheless, because complication rates continue to be reported as low, IO use should be considered early on for urgent vascular access.

The study was not without limitations. The exact temporal relationship between IO placement and outcomes is unknown due to constraints within the TriNetX database, which only allows users to determine when CPT and ICD codes are billed. We can only assume that the patient receives IO placement and is diagnosed on the same day they are billed. Additionally, we would have expected a higher mortality rate among this population due to the likely prognosis of critically ill patients receiving IO placement. Therefore, it is possible that not all IO placement codes were recorded correctly within the EHR, or some procedures took place but were not documented at all. It should also be considered that IO procedural codes are not necessarily recorded for missed attempts. In our study, the location of IO placement for 40% of the encounters was not reported. It is possible that the patient received an IO line in a clinical setting that was not the emergency department or inpatient area. Even though it was unknown where these encounters occurred, we opted to include these subjects as IOs are typically utilized as an emergency tool in the ED or inpatient setting. Additionally, we must consider that patients who survived in a hospital represented within the TriNetX database may not have survived in a non-TriNetX hospital if they were transferred out of the TriNetX hospital. Due to dataset limitations, the severity of the illness was unknown. However, the patient population was likely critically ill due to IO usage in emergencies. Furthermore, the data was de-identified such that no information was provided about the hospitals within the database (including whether they were pediatric-based hospitals). Despite these limitations, the data set provided a large sample size using population-based sampling of 37 different HCOs across the USA.

## Conclusion

In conclusion, we found that more IOs were placed in subjects ≥ 1 year of age, and a higher mortality rate was seen in subjects < 1 year of age. Lower frequencies of noncardiac diagnoses at the time of IO placement were found in both groups, highlighting IO may be underutilized in noncardiac settings such as convulsions, shock, and respiratory failure. Given the low rate of complications seen in both groups of our study, IO use should be considered early for urgent vascular access, especially for children less than 1 year of age.

## Supplementary Information

**Additional file 1: Supplemental Table 1.** Diagnoses Evaluated During Time Period of Intraosseous Access Procedural Code.

## Data Availability

The datasets generated and/or analyzed during the current study are not publicly available but are available from the corresponding author on reasonable request.

## Abbreviations

IO: Intraosseous
AHA PALS: American Heart Association’s Pediatric Advanced Life Support
EHR: Electronic health record
HCO: Healthcare organization
CPT: Common procedural terminology
IRB: Institutional Review Board
HIPAA: Health Insurance Portability and Accountability Act
ISMS: Information security management system
ICD: International Classification of Diseases
ED: Emergency department
IM: Intramuscular
PICU: Pediatric intensive care unit
