# Supplementary material for: Current utilization of interosseous access in pediatrics: a population-based analysis using an EHR database, TriNetX
Source: Int J Emerg Med. 2022 Nov 29;15:65. doi: 10.1186/s12245-022-00467-9 (PMC9706868; doi:10.1186/s12245-022-00467-9)
Supplement: Supplementary file 1 — Additional file 1: Supplemental Table 1. Diagnoses Evaluated During Time Period of Intraosseous Access Procedural Code. [file 12245_2022_467_MOESM1_ESM.docx]

| Supplemental Table 1: Diagnoses Evaluated During Time Period of Intraosseous Access Procedural Code | |
| --- | --- |
| Cardiac Arrest | "427.5" (ICD-9-CM: "Cardiac arrest"); "427.41" (ICD-9-CM: "Ventricular fibrillation"); "I46.9" (ICD-10-CM: "Cardiac arrest, cause unspecified"); "I46.8" (ICD-10-CM: "Cardiac arrest due to other underlying condition"); "I46.2" (ICD-10-CM: "Cardiac arrest due to underlying cardiac condition"); "Z86.74" (ICD-10-CM: "Personal history of sudden cardiac arrest"); "I49.01" (ICD-10-CM: "Ventricular fibrillation") |
| Convulsions | "345.3" (ICD-9-CM: "Grand mal status"); "780.39" (ICD-9-CM: "Other convulsions"); "780.32" (ICD-9-CM: "Complex febrile convulsions"); "345.1" (ICD-9-CM: "Generalized convulsive epilepsy, without mention of intractable epilepsy"); "345.11" (ICD-9-CM: "Generalized convulsive epilepsy, with intractable epilepsy"); "345.4" (ICD-9-CM: "Localization-related (focal) (partial) epilepsy and epileptic syndromes with complex partial seizures, without mention of intractable epilepsy"); "345.9" (ICD-9-CM: "Epilepsy, unspecified, without mention of intractable epilepsy"); "780.33" (ICD-9-CM: "Post traumatic seizures"); "345.91" (ICD-9-CM: "Epilepsy, unspecified, with intractable epilepsy"); "345.8" (ICD-9-CM: "Other forms of epilepsy and recurrent seizures, without mention of intractable epilepsy"); "345.7" (ICD-9-CM: "Epilepsia partialis continua, without mention of intractable epilepsy"); "780.31" (ICD-9-CM: "Febrile convulsions (simple), unspecified"); "345.41" (ICD-9-CM: "Localization-related (focal) (partial) epilepsy and epileptic syndromes with complex partial seizures, with intractable epilepsy"); "345.61" (ICD-9-CM: "Infantile spasms, with intractable epilepsy"); "345.5" (ICD-9-CM: "Localization-related (focal) (partial) epilepsy and epileptic syndromes with simple partial seizures, without mention of intractable epilepsy"); "345.51" (ICD-9-CM: "Localization-related (focal) (partial) epilepsy and epileptic syndromes with simple partial seizures, with intractable epilepsy"); "345.6" (ICD-9-CM: "Infantile spasms, without mention of intractable epilepsy"); "G40.901" (ICD-10-CM: "Epilepsy, unspecified, not intractable, with status epilepticus"); "R56.01" (ICD-10-CM: "Complex febrile convulsions"); "R56.9" (ICD-10-CM: "Unspecified convulsions"); "G40.409" (ICD-10-CM: "Other generalized epilepsy and epileptic syndromes, not intractable, without status epilepticus"); "G40.802" (ICD-10-CM: "Other epilepsy, not intractable, without status epilepticus"); "G40.909" (ICD-10-CM: "Epilepsy, unspecified, not intractable, without status epilepticus"); "G40.211" (ICD-10-CM: "Localization-related (focal) (partial) symptomatic epilepsy and epileptic syndromes with complex partial seizures, intractable, with status epilepticus"); "Z82.0" (ICD-10-CM: "Family history of epilepsy and other diseases of the nervous system"); "G40.109" (ICD-10-CM: "Localization-related (focal) (partial) symptomatic epilepsy and epileptic syndromes with simple partial seizures, not intractable, without status epilepticus"); "G40.822" (ICD-10-CM: "Epileptic spasms, not intractable, without status epilepticus"); "G40.824" (ICD-10-CM: "Epileptic spasms, intractable, without status epilepticus"); "R56.00" (ICD-10-CM: "Simple febrile convulsions"); "G40.301" (ICD-10-CM: "Generalized idiopathic epilepsy and epileptic syndromes, not intractable, with status epilepticus"); "G40.411" (ICD-10-CM: "Other generalized epilepsy and epileptic syndromes, intractable, with status epilepticus"); "G40.814" (ICD-10-CM: "Lennox-Gastaut syndrome, intractable, without status epilepticus"); "G40.919" (ICD-10-CM: "Epilepsy, unspecified, intractable, without status epilepticus"); "G40.119" (ICD-10-CM: "Localization-related (focal) (partial) symptomatic epilepsy and epileptic syndromes with simple partial seizures, intractable, without status epilepticus"); "G40.911" (ICD-10-CM: "Epilepsy, unspecified, intractable, with status epilepticus"); "G40.401" (ICD-10-CM: "Other generalized epilepsy and epileptic syndromes, not intractable, with status epilepticus"); "G40.219" (ICD-10-CM: "Localization-related (focal) (partial) symptomatic epilepsy and epileptic syndromes with complex partial seizures, intractable, without status epilepticus"); "R56.1" (ICD-10-CM: "Post traumatic seizures"); "G40.813" (ICD-10-CM: "Lennox-Gastaut syndrome, intractable, with status epilepticus"); "G40.201" (ICD-10-CM: "Localization-related (focal) (partial) symptomatic epilepsy and epileptic syndromes with complex partial seizures, not intractable, with status epilepticus"); "G40.101" (ICD-10-CM: "Localization-related (focal) (partial) symptomatic epilepsy and epileptic syndromes with simple partial seizures, not intractable, with status epilepticus"); "G40.89" (ICD-10-CM: "Other seizures"); "G40.209" (ICD-10-CM: "Localization-related (focal) (partial) symptomatic epilepsy and epileptic syndromes with complex partial seizures, not intractable, without status epilepticus"); "G40.801" (ICD-10-CM: "Other epilepsy, not intractable, with status epilepticus"); "G40.804" (ICD-10-CM: "Other epilepsy, intractable, without status epilepticus"); "G40.319" (ICD-10-CM: "Generalized idiopathic epilepsy and epileptic syndromes, intractable, without status epilepticus"); "G40.419" (ICD-10-CM: "Other generalized epilepsy and epileptic syndromes, intractable, without status epilepticus"); "G40.309" (ICD-10-CM: "Generalized idiopathic epilepsy and epileptic syndromes, not intractable, without status epilepticus"); "G40.823" (ICD-10-CM: "Epileptic spasms, intractable, with status epilepticus"); "G83.84" (ICD-10-CM: "Todd's paralysis (postepileptic)") |
| Respiratory Failure | "518.81" (ICD-9-CM: "Acute respiratory failure"); "518.83" (ICD-9-CM: "Chronic respiratory failure"); "518.51" (ICD-9-CM: "Acute respiratory failure following trauma and surgery"); "518.84" (ICD-9-CM: "Acute and chronic respiratory failure"); "J96.01" (ICD-10-CM: "Acute respiratory failure with hypoxia"); "J96.90" (ICD-10-CM: "Respiratory failure, unspecified, unspecified whether with hypoxia or hypercapnia"); "J96.00" (ICD-10-CM: "Acute respiratory failure, unspecified whether with hypoxia or hypercapnia"); "J96.02" (ICD-10-CM: "Acute respiratory failure with hypercapnia"); "J96.91" (ICD-10-CM: "Respiratory failure, unspecified with hypoxia"); "J96.21" (ICD-10-CM: "Acute and chronic respiratory failure with hypoxia"); "J96.22" (ICD-10-CM: "Acute and chronic respiratory failure with hypercapnia"); "J96.10" (ICD-10-CM: "Chronic respiratory failure, unspecified whether with hypoxia or hypercapnia"); "J95.821" (ICD-10-CM: "Acute postprocedural respiratory failure"); "J96.11" (ICD-10-CM: "Chronic respiratory failure with hypoxia"); "J96.12" (ICD-10-CM: "Chronic respiratory failure with hypercapnia"); "J96.20" (ICD-10-CM: "Acute and chronic respiratory failure, unspecified whether with hypoxia or hypercapnia"); "J96.92" (ICD-10-CM: "Respiratory failure, unspecified with hypercapnia") |
| Shock | "276.51" (ICD-9-CM: "Dehydration"); "276.52" (ICD-9-CM: "Hypovolemia"); "785.5" (ICD-9-CM: "Shock, unspecified"); "790.7" (ICD-9-CM: "Bacteremia"); "458.9" (ICD-9-CM: "Hypotension, unspecified"); "785.59" (ICD-9-CM: "Other shock without mention of trauma"); "785.52" (ICD-9-CM: "Septic shock"); "785.51" (ICD-9-CM: "Cardiogenic shock"); "458.8" (ICD-9-CM: "Other specified hypotension"); "958.4" (ICD-9-CM: "Traumatic shock"); "40.82" (ICD-9-CM: "Toxic shock syndrome"); "458.29" (ICD-9-CM: "Other iatrogenic hypotension"); "E86.0" (ICD-10-CM: "Dehydration"); "I95.9" (ICD-10-CM: "Hypotension, unspecified"); "R57.9" (ICD-10-CM: "Shock, unspecified"); "R57.8" (ICD-10-CM: "Other shock"); "R57.1" (ICD-10-CM: "Hypovolemic shock"); "R78.81" (ICD-10-CM: "Bacteremia"); "T79.4XXA" (ICD-10-CM: "Traumatic shock, initial encounter"); "E86.1" (ICD-10-CM: "Hypovolemia"); "R65.21" (ICD-10-CM: "Severe sepsis with septic shock"); "R57.0" (ICD-10-CM: "Cardiogenic shock"); "R65.20" (ICD-10-CM: "Severe sepsis without septic shock"); "I95.89" (ICD-10-CM: "Other hypotension"); "I95.81" (ICD-10-CM: "Postprocedural hypotension"); "I95.2" (ICD-10-CM: "Hypotension due to drugs"); "T81.19XA" (ICD-10-CM: "Other postprocedural shock, initial encounter") |
| Certain infectious and parasitic diseases | "38.9" (ICD-9-CM: "Unspecified septicemia"); "995.91" (ICD-9-CM: "Sepsis"); "41.3" (ICD-9-CM: "FriedlÃƒÂ¤nder's bacillus infection in conditions classified elsewhere and of unspecified site"); "79.99" (ICD-9-CM: "Unspecified viral infection"); "54.9" (ICD-9-CM: "Herpes simplex without mention of complication"); "79.6" (ICD-9-CM: "Respiratory syncytial virus (RSV)"); "40.41" (ICD-9-CM: "Infant botulism"); "41.7" (ICD-9-CM: "Pseudomonas infection in conditions classified elsewhere and of unspecified site"); "41.85" (ICD-9-CM: "Other specified bacterial infections in conditions classified elsewhere and of unspecified site, other gram-negative organisms"); "79.3" (ICD-9-CM: "Rhinovirus infection in conditions classified elsewhere and of unspecified site"); "41.09" (ICD-9-CM: "Streptococcus infection in conditions classified elsewhere and of unspecified site, other streptococcus"); "79.89" (ICD-9-CM: "Other specified viral infection"); "38.2" (ICD-9-CM: "Pneumococcal septicemia [Streptococcus pneumoniae septicemia]"); "41.12" (ICD-9-CM: "Methicillin resistant Staphylococcus aureus in conditions classified elsewhere and of unspecified site"); "8.8" (ICD-9-CM: "Intestinal infection due to other organism, not elsewhere classified"); "8.63" (ICD-9-CM: "Enteritis due to norwalk virus"); "41.11" (ICD-9-CM: "Methicillin susceptible Staphylococcus aureus in conditions classified elsewhere and of unspecified site"); "8.45" (ICD-9-CM: "Intestinal infection due to Clostridium difficile"); "9" (ICD-9-CM: "Infectious colitis, enteritis, and gastroenteritis"); "36" (ICD-9-CM: "Meningococcal meningitis"); "36.2" (ICD-9-CM: "Meningococcemia"); "79" (ICD-9-CM: "Adenovirus infection in conditions classified elsewhere and of unspecified site"); "101" (ICD-9-CM: "Vincent's angina"); "38" (ICD-9-CM: "Streptococcal septicemia"); "40.82" (ICD-9-CM: "Toxic shock syndrome"); "41.01" (ICD-9-CM: "Streptococcus infection in conditions classified elsewhere and of unspecified site, streptococcus, group A"); "112" (ICD-9-CM: "Candidiasis of mouth"); "38.43" (ICD-9-CM: "Septicemia due to pseudomonas"); "41.49" (ICD-9-CM: "Other and unspecified Escherichia coli [E. coli]"); "38.4" (ICD-9-CM: "Septicemia due to gram-negative organism, unspecified"); "38.11" (ICD-9-CM: "Methicillin susceptible Staphylococcus aureus septicemia"); "112.2" (ICD-9-CM: "Candidiasis of other urogenital sites"); "47.9" (ICD-9-CM: "Unspecified viral meningitis"); "B97.4" (ICD-10-CM: "Respiratory syncytial virus as the cause of diseases classified elsewhere"); "A41.9" (ICD-10-CM: "Sepsis, unspecified organism"); "A41.81" (ICD-10-CM: "Sepsis due to Enterococcus"); "A41.1" (ICD-10-CM: "Sepsis due to other specified staphylococcus"); "B37.9" (ICD-10-CM: "Candidiasis, unspecified"); "B95.7" (ICD-10-CM: "Other staphylococcus as the cause of diseases classified elsewhere"); "B34.0" (ICD-10-CM: "Adenovirus infection, unspecified"); "A08.11" (ICD-10-CM: "Acute gastroenteropathy due to Norwalk agent"); "B96.4" (ICD-10-CM: "Proteus (mirabilis) (morganii) as the cause of diseases classified elsewhere"); "B49" (ICD-10-CM: "Unspecified mycosis"); "B34.8" (ICD-10-CM: "Other viral infections of unspecified site"); "B97.89" (ICD-10-CM: "Other viral agents as the cause of diseases classified elsewhere"); "B96.5" (ICD-10-CM: "Pseudomonas (aeruginosa) (mallei) (pseudomallei) as the cause of diseases classified elsewhere"); "A08.4" (ICD-10-CM: "Viral intestinal infection, unspecified"); "B95.3" (ICD-10-CM: "Streptococcus pneumoniae as the cause of diseases classified elsewhere"); "A40.3" (ICD-10-CM: "Sepsis due to Streptococcus pneumoniae"); "B95.62" (ICD-10-CM: "Methicillin resistant Staphylococcus aureus infection as the cause of diseases classified elsewhere"); "B96.1" (ICD-10-CM: "Klebsiella pneumoniae [K. pneumoniae] as the cause of diseases classified elsewhere"); "B96.89" (ICD-10-CM: "Other specified bacterial agents as the cause of diseases classified elsewhere"); "B97.81" (ICD-10-CM: "Human metapneumovirus as the cause of diseases classified elsewhere"); "A04.4" (ICD-10-CM: "Other intestinal Escherichia coli infections"); "B96.20" (ICD-10-CM: "Unspecified Escherichia coli [E. coli] as the cause of diseases classified elsewhere"); "A41.89" (ICD-10-CM: "Other specified sepsis"); "A41.51" (ICD-10-CM: "Sepsis due to Escherichia coli [E. coli]"); "B37.0" (ICD-10-CM: "Candidal stomatitis"); "B97.0" (ICD-10-CM: "Adenovirus as the cause of diseases classified elsewhere"); "B37.2" (ICD-10-CM: "Candidiasis of skin and nail"); "B37.49" (ICD-10-CM: "Other urogenital candidiasis"); "B95.61" (ICD-10-CM: "Methicillin susceptible Staphylococcus aureus infection as the cause of diseases classified elsewhere"); "A87.9" (ICD-10-CM: "Viral meningitis, unspecified"); "A02.1" (ICD-10-CM: "Salmonella sepsis"); "B34.1" (ICD-10-CM: "Enterovirus infection, unspecified"); "A08.0" (ICD-10-CM: "Rotaviral enteritis"); "B96.3" (ICD-10-CM: "Hemophilus influenzae [H. influenzae] as the cause of diseases classified elsewhere"); "A09" (ICD-10-CM: "Infectious gastroenteritis and colitis, unspecified"); "A04.72" (ICD-10-CM: "Enterocolitis due to Clostridium difficile, not specified as recurrent"); "A08.32" (ICD-10-CM: "Astrovirus enteritis"); "A08.39" (ICD-10-CM: "Other viral enteritis"); "B97.19" (ICD-10-CM: "Other enterovirus as the cause of diseases classified elsewhere"); "B34.9" (ICD-10-CM: "Viral infection, unspecified"); "A41.52" (ICD-10-CM: "Sepsis due to Pseudomonas"); "B97.29" (ICD-10-CM: "Other coronavirus as the cause of diseases classified elsewhere"); "A41.01" (ICD-10-CM: "Sepsis due to Methicillin susceptible Staphylococcus aureus"); "B95.5" (ICD-10-CM: "Unspecified streptococcus as the cause of diseases classified elsewhere"); "A85.0" (ICD-10-CM: "Enteroviral encephalitis"); "B97.10" (ICD-10-CM: "Unspecified enterovirus as the cause of diseases classified elsewhere"); "B33.8" (ICD-10-CM: "Other specified viral diseases"); "B34.2" (ICD-10-CM: "Coronavirus infection, unspecified"); "A49.8" (ICD-10-CM: "Other bacterial infections of unspecified site"); "B37.3" (ICD-10-CM: "Candidiasis of vulva and vagina"); "B99.9" (ICD-10-CM: "Unspecified infectious disease"); "B95.2" (ICD-10-CM: "Enterococcus as the cause of diseases classified elsewhere"); "B08.1" (ICD-10-CM: "Molluscum contagiosum"); "B95.1" (ICD-10-CM: "Streptococcus, group B, as the cause of diseases classified elsewhere"); "A41.59" (ICD-10-CM: "Other Gram-negative sepsis"); "A49.9" (ICD-10-CM: "Bacterial infection, unspecified"); "A87.0" (ICD-10-CM: "Enteroviral meningitis"); "B00.3" (ICD-10-CM: "Herpesviral meningitis"); "B10.81" (ICD-10-CM: "Human herpesvirus 6 infection"); "A49.02" (ICD-10-CM: "Methicillin resistant Staphylococcus aureus infection, unspecified site"); "A48.8" (ICD-10-CM: "Other specified bacterial diseases"); "B94.8" (ICD-10-CM: "Sequelae of other specified infectious and parasitic diseases"); "A68.9" (ICD-10-CM: "Relapsing fever, unspecified"); "A49.01" (ICD-10-CM: "Methicillin susceptible Staphylococcus aureus infection, unspecified site"); "A49.1" (ICD-10-CM: "Streptococcal infection, unspecified site") |
| Diseases of the nervous system | "345.3" (ICD-9-CM: "Grand mal status"); "343.2" (ICD-9-CM: "Congenital quadriplegia"); "342.91" (ICD-9-CM: "Hemiplegia, unspecified, affecting dominant side"); "348.4" (ICD-9-CM: "Compression of brain"); "348.5" (ICD-9-CM: "Cerebral edema"); "348.1" (ICD-9-CM: "Anoxic brain damage"); "343.9" (ICD-9-CM: "Infantile cerebral palsy, unspecified"); "345.1" (ICD-9-CM: "Generalized convulsive epilepsy, without mention of intractable epilepsy"); "345.11" (ICD-9-CM: "Generalized convulsive epilepsy, with intractable epilepsy"); "345.4" (ICD-9-CM: "Localization-related (focal) (partial) epilepsy and epileptic syndromes with complex partial seizures, without mention of intractable epilepsy"); "345.9" (ICD-9-CM: "Epilepsy, unspecified, without mention of intractable epilepsy"); "325" (ICD-9-CM: "Phlebitis and thrombophlebitis of intracranial venous sinuses"); "348.82" (ICD-9-CM: "Brain death"); "333.2" (ICD-9-CM: "Myoclonus"); "327.23" (ICD-9-CM: "Obstructive sleep apnea (adult)(pediatric)"); "348.3" (ICD-9-CM: "Encephalopathy, unspecified"); "344.89" (ICD-9-CM: "Other specified paralytic syndrome"); "780.59" (ICD-9-CM: "Other sleep disturbances"); "331.4" (ICD-9-CM: "Obstructive hydrocephalus"); "345.91" (ICD-9-CM: "Epilepsy, unspecified, with intractable epilepsy"); "327.24" (ICD-9-CM: "Idiopathic sleep related non-obstructive alveolar hypoventilation"); "780.57" (ICD-9-CM: "Unspecified sleep apnea"); "338.11" (ICD-9-CM: "Acute pain due to trauma"); "780.54" (ICD-9-CM: "Hypersomnia, unspecified"); "345.8" (ICD-9-CM: "Other forms of epilepsy and recurrent seizures, without mention of intractable epilepsy"); "335" (ICD-9-CM: "Werdnig-Hoffmann disease"); "322.9" (ICD-9-CM: "Meningitis, unspecified"); "320.1" (ICD-9-CM: "Pneumococcal meningitis"); "320.2" (ICD-9-CM: "Streptococcal meningitis"); "345.7" (ICD-9-CM: "Epilepsia partialis continua, without mention of intractable epilepsy"); "348" (ICD-9-CM: "Cerebral cysts"); "344" (ICD-9-CM: "Quadriplegia, unspecified"); "348.9" (ICD-9-CM: "Unspecified condition of brain"); "348.8" (ICD-9-CM: "Other conditions of brain"); "348.31" (ICD-9-CM: "Metabolic encephalopathy"); "348.89" (ICD-9-CM: "Other conditions of brain"); "342.92" (ICD-9-CM: "Hemiplegia, unspecified, affecting nondominant side"); "330.8" (ICD-9-CM: "Other specified cerebral degenerations in childhood"); "348.2" (ICD-9-CM: "Benign intracranial hypertension"); "351" (ICD-9-CM: "Bell's palsy"); "345.41" (ICD-9-CM: "Localization-related (focal) (partial) epilepsy and epileptic syndromes with complex partial seizures, with intractable epilepsy"); "345.61" (ICD-9-CM: "Infantile spasms, with intractable epilepsy"); "345.5" (ICD-9-CM: "Localization-related (focal) (partial) epilepsy and epileptic syndromes with simple partial seizures, without mention of intractable epilepsy"); "327.26" (ICD-9-CM: "Sleep related hypoventilation/hypoxemia in conditions classifiable elsewhere"); "345.51" (ICD-9-CM: "Localization-related (focal) (partial) epilepsy and epileptic syndromes with simple partial seizures, with intractable epilepsy"); "345.6" (ICD-9-CM: "Infantile spasms, without mention of intractable epilepsy"); "G40.901" (ICD-10-CM: "Epilepsy, unspecified, not intractable, with status epilepticus"); "G40.409" (ICD-10-CM: "Other generalized epilepsy and epileptic syndromes, not intractable, without status epilepticus"); "G40.802" (ICD-10-CM: "Other epilepsy, not intractable, without status epilepticus"); "G40.909" (ICD-10-CM: "Epilepsy, unspecified, not intractable, without status epilepticus"); "G93.2" (ICD-10-CM: "Benign intracranial hypertension"); "G93.6" (ICD-10-CM: "Cerebral edema"); "G93.40" (ICD-10-CM: "Encephalopathy, unspecified"); "G93.41" (ICD-10-CM: "Metabolic encephalopathy"); "G40.211" (ICD-10-CM: "Localization-related (focal) (partial) symptomatic epilepsy and epileptic syndromes with complex partial seizures, intractable, with status epilepticus"); "G10" (ICD-10-CM: "Huntington's disease"); "G90.1" (ICD-10-CM: "Familial dysautonomia [Riley-Day]"); "G93.49" (ICD-10-CM: "Other encephalopathy"); "G91.9" (ICD-10-CM: "Hydrocephalus, unspecified"); "G60.0" (ICD-10-CM: "Hereditary motor and sensory neuropathy"); "G80.9" (ICD-10-CM: "Cerebral palsy, unspecified"); "G40.109" (ICD-10-CM: "Localization-related (focal) (partial) symptomatic epilepsy and epileptic syndromes with simple partial seizures, not intractable, without status epilepticus"); "G40.822" (ICD-10-CM: "Epileptic spasms, not intractable, without status epilepticus"); "G40.824" (ICD-10-CM: "Epileptic spasms, intractable, without status epilepticus"); "G40.301" (ICD-10-CM: "Generalized idiopathic epilepsy and epileptic syndromes, not intractable, with status epilepticus"); "G40.411" (ICD-10-CM: "Other generalized epilepsy and epileptic syndromes, intractable, with status epilepticus"); "G93.1" (ICD-10-CM: "Anoxic brain damage, not elsewhere classified"); "G03.9" (ICD-10-CM: "Meningitis, unspecified"); "G93.5" (ICD-10-CM: "Compression of brain"); "G47.00" (ICD-10-CM: "Insomnia, unspecified"); "G47.33" (ICD-10-CM: "Obstructive sleep apnea (adult) (pediatric)"); "G80.0" (ICD-10-CM: "Spastic quadriplegic cerebral palsy"); "G40.814" (ICD-10-CM: "Lennox-Gastaut syndrome, intractable, without status epilepticus"); "G40.919" (ICD-10-CM: "Epilepsy, unspecified, intractable, without status epilepticus"); "G93.89" (ICD-10-CM: "Other specified disorders of brain"); "G90.8" (ICD-10-CM: "Other disorders of autonomic nervous system"); "G40.119" (ICD-10-CM: "Localization-related (focal) (partial) symptomatic epilepsy and epileptic syndromes with simple partial seizures, intractable, without status epilepticus"); "G82.50" (ICD-10-CM: "Quadriplegia, unspecified"); "G90.4" (ICD-10-CM: "Autonomic dysreflexia"); "G80.1" (ICD-10-CM: "Spastic diplegic cerebral palsy"); "G40.911" (ICD-10-CM: "Epilepsy, unspecified, intractable, with status epilepticus"); "G00.1" (ICD-10-CM: "Pneumococcal meningitis"); "G91.8" (ICD-10-CM: "Other hydrocephalus"); "G25.3" (ICD-10-CM: "Myoclonus"); "G12.0" (ICD-10-CM: "Infantile spinal muscular atrophy, type I [Werdnig-Hoffman]"); "G40.401" (ICD-10-CM: "Other generalized epilepsy and epileptic syndromes, not intractable, with status epilepticus"); "G93.82" (ICD-10-CM: "Brain death"); "G40.219" (ICD-10-CM: "Localization-related (focal) (partial) symptomatic epilepsy and epileptic syndromes with complex partial seizures, intractable, without status epilepticus"); "G95.0" (ICD-10-CM: "Syringomyelia and syringobulbia"); "G47.34" (ICD-10-CM: "Idiopathic sleep related nonobstructive alveolar hypoventilation"); "G96.0" (ICD-10-CM: "Cerebrospinal fluid leak"); "G96.9" (ICD-10-CM: "Disorder of central nervous system, unspecified"); "G70.9" (ICD-10-CM: "Myoneural disorder, unspecified"); "G62.81" (ICD-10-CM: "Critical illness polyneuropathy"); "G91.0" (ICD-10-CM: "Communicating hydrocephalus"); "G40.813" (ICD-10-CM: "Lennox-Gastaut syndrome, intractable, with status epilepticus"); "G40.201" (ICD-10-CM: "Localization-related (focal) (partial) symptomatic epilepsy and epileptic syndromes with complex partial seizures, not intractable, with status epilepticus"); "G89.11" (ICD-10-CM: "Acute pain due to trauma"); "G40.101" (ICD-10-CM: "Localization-related (focal) (partial) symptomatic epilepsy and epileptic syndromes with simple partial seizures, not intractable, with status epilepticus"); "G80.8" (ICD-10-CM: "Other cerebral palsy"); "G90.9" (ICD-10-CM: "Disorder of the autonomic nervous system, unspecified"); "G12.9" (ICD-10-CM: "Spinal muscular atrophy, unspecified"); "G89.18" (ICD-10-CM: "Other acute postprocedural pain"); "G12.29" (ICD-10-CM: "Other motor neuron disease"); "G25.5" (ICD-10-CM: "Other chorea"); "G04.90" (ICD-10-CM: "Encephalitis and encephalomyelitis, unspecified"); "G47.30" (ICD-10-CM: "Sleep apnea, unspecified"); "G89.29" (ICD-10-CM: "Other chronic pain"); "G40.89" (ICD-10-CM: "Other seizures"); "G40.209" (ICD-10-CM: "Localization-related (focal) (partial) symptomatic epilepsy and epileptic syndromes with complex partial seizures, not intractable, without status epilepticus"); "G00.2" (ICD-10-CM: "Streptococcal meningitis"); "G00.9" (ICD-10-CM: "Bacterial meningitis, unspecified"); "G97.82" (ICD-10-CM: "Other postprocedural complications and disorders of nervous system"); "G93.9" (ICD-10-CM: "Disorder of brain, unspecified"); "G40.801" (ICD-10-CM: "Other epilepsy, not intractable, with status epilepticus"); "G40.804" (ICD-10-CM: "Other epilepsy, intractable, without status epilepticus"); "G47.36" (ICD-10-CM: "Sleep related hypoventilation in conditions classified elsewhere"); "G81.91" (ICD-10-CM: "Hemiplegia, unspecified affecting right dominant side"); "G25.2" (ICD-10-CM: "Other specified forms of tremor"); "G47.8" (ICD-10-CM: "Other sleep disorders"); "G93.0" (ICD-10-CM: "Cerebral cysts"); "G40.319" (ICD-10-CM: "Generalized idiopathic epilepsy and epileptic syndromes, intractable, without status epilepticus"); "G47.9" (ICD-10-CM: "Sleep disorder, unspecified"); "G31.9" (ICD-10-CM: "Degenerative disease of nervous system, unspecified"); "G62.9" (ICD-10-CM: "Polyneuropathy, unspecified"); "G52.7" (ICD-10-CM: "Disorders of multiple cranial nerves"); "G25.9" (ICD-10-CM: "Extrapyramidal and movement disorder, unspecified"); "G71.0" (ICD-10-CM: "Muscular dystrophy"); "G71.2" (ICD-10-CM: "Congenital myopathies"); "G72.81" (ICD-10-CM: "Critical illness myopathy"); "G25.1" (ICD-10-CM: "Drug-induced tremor"); "G40.419" (ICD-10-CM: "Other generalized epilepsy and epileptic syndromes, intractable, without status epilepticus"); "G71.3" (ICD-10-CM: "Mitochondrial myopathy, not elsewhere classified"); "G40.309" (ICD-10-CM: "Generalized idiopathic epilepsy and epileptic syndromes, not intractable, without status epilepticus"); "G40.823" (ICD-10-CM: "Epileptic spasms, intractable, with status epilepticus"); "G80.2" (ICD-10-CM: "Spastic hemiplegic cerebral palsy"); "G83.84" (ICD-10-CM: "Todd's paralysis (postepileptic)"); "G96.08" (ICD-10-CM: "Other cranial cerebrospinal fluid leak"); "G92" (ICD-10-CM: "Toxic encephalopathy") |
| Diseases of the respiratory system | "518.81" (ICD-9-CM: "Acute respiratory failure"); "518.82" (ICD-9-CM: "Other pulmonary insufficiency, not elsewhere classified"); "519.8" (ICD-9-CM: "Other diseases of respiratory system, not elsewhere classified"); "464.4" (ICD-9-CM: "Croup"); "486" (ICD-9-CM: "Pneumonia, organism unspecified"); "493.9" (ICD-9-CM: "Asthma, unspecified type, unspecified"); "518.83" (ICD-9-CM: "Chronic respiratory failure"); "507" (ICD-9-CM: "Pneumonitis due to inhalation of food or vomitus"); "518" (ICD-9-CM: "Pulmonary collapse"); "518.89" (ICD-9-CM: "Other diseases of lung, not elsewhere classified"); "465.9" (ICD-9-CM: "Acute upper respiratory infections of unspecified site"); "466.11" (ICD-9-CM: "Acute bronchiolitis due to respiratory syncytial virus (RSV)"); "512.89" (ICD-9-CM: "Other pneumothorax"); "480.8" (ICD-9-CM: "Pneumonia due to other virus not elsewhere classified"); "482.1" (ICD-9-CM: "Pneumonia due to Pseudomonas"); "512.8" (ICD-9-CM: "Other pneumothorax and air leak"); "464.1" (ICD-9-CM: "Acute tracheitis without mention of obstruction"); "511.89" (ICD-9-CM: "Other specified forms of effusion, except tuberculous"); "466" (ICD-9-CM: "Acute bronchitis"); "514" (ICD-9-CM: "Pulmonary congestion and hypostasis"); "496" (ICD-9-CM: "Chronic airway obstruction, not elsewhere classified"); "478.6" (ICD-9-CM: "Edema of larynx"); "518.51" (ICD-9-CM: "Acute respiratory failure following trauma and surgery"); "518.84" (ICD-9-CM: "Acute and chronic respiratory failure"); "482.9" (ICD-9-CM: "Bacterial pneumonia, unspecified"); "518.4" (ICD-9-CM: "Acute edema of lung, unspecified"); "519.11" (ICD-9-CM: "Acute bronchospasm"); "483.8" (ICD-9-CM: "Pneumonia due to other specified organism"); "477.9" (ICD-9-CM: "Allergic rhinitis, cause unspecified"); "519.19" (ICD-9-CM: "Other diseases of trachea and bronchus"); "478.34" (ICD-9-CM: "Bilateral paralysis of vocal cords or larynx, complete"); "493.92" (ICD-9-CM: "Asthma, unspecified type, with (acute) exacerbation"); "478.19" (ICD-9-CM: "Other disease of nasal cavity and sinuses"); "508.2" (ICD-9-CM: "Respiratory conditions due to smoke inhalation"); "482" (ICD-9-CM: "Pneumonia due to Klebsiella pneumoniae"); "511.9" (ICD-9-CM: "Unspecified pleural effusion"); "466.19" (ICD-9-CM: "Acute bronchiolitis due to other infectious organisms"); "518.3" (ICD-9-CM: "Pulmonary eosinophilia"); "480.1" (ICD-9-CM: "Pneumonia due to respiratory syncytial virus"); "34" (ICD-9-CM: "Streptococcal sore throat"); "482.31" (ICD-9-CM: "Pneumonia due to Streptococcus, group A"); "481" (ICD-9-CM: "Pneumococcal pneumonia [Streptococcus pneumoniae pneumonia]"); "519.1" (ICD-9-CM: "Other diseases of trachea and bronchus, not elsewhere classified"); "518.5" (ICD-9-CM: "Pulmonary insufficiency following trauma and surgery"); "478.74" (ICD-9-CM: "Stenosis of larynx"); "518.52" (ICD-9-CM: "Other pulmonary insufficiency, not elsewhere classified, following trauma and surgery"); "487" (ICD-9-CM: "Influenza with pneumonia"); "492.8" (ICD-9-CM: "Other emphysema"); "493.91" (ICD-9-CM: "Asthma, unspecified type, with status asthmaticus"); "478.31" (ICD-9-CM: "Unilateral paralysis of vocal cords or larynx, partial"); "476" (ICD-9-CM: "Chronic laryngitis"); "519.09" (ICD-9-CM: "Other tracheostomy complications"); "519.9" (ICD-9-CM: "Unspecified disease of respiratory system"); "J06.9" (ICD-10-CM: "Acute upper respiratory infection, unspecified"); "J18.9" (ICD-10-CM: "Pneumonia, unspecified organism"); "J18.1" (ICD-10-CM: "Lobar pneumonia, unspecified organism"); "J96.01" (ICD-10-CM: "Acute respiratory failure with hypoxia"); "J12.1" (ICD-10-CM: "Respiratory syncytial virus pneumonia"); "J21.0" (ICD-10-CM: "Acute bronchiolitis due to respiratory syncytial virus"); "J96.90" (ICD-10-CM: "Respiratory failure, unspecified, unspecified whether with hypoxia or hypercapnia"); "J96.00" (ICD-10-CM: "Acute respiratory failure, unspecified whether with hypoxia or hypercapnia"); "J15.8" (ICD-10-CM: "Pneumonia due to other specified bacteria"); "J45.909" (ICD-10-CM: "Unspecified asthma, uncomplicated"); "J04.10" (ICD-10-CM: "Acute tracheitis without obstruction"); "J95.09" (ICD-10-CM: "Other tracheostomy complication"); "J98.9" (ICD-10-CM: "Respiratory disorder, unspecified"); "J45.902" (ICD-10-CM: "Unspecified asthma with status asthmaticus"); "J80" (ICD-10-CM: "Acute respiratory distress syndrome"); "J90" (ICD-10-CM: "Pleural effusion, not elsewhere classified"); "J96.02" (ICD-10-CM: "Acute respiratory failure with hypercapnia"); "J98.11" (ICD-10-CM: "Atelectasis"); "J69.0" (ICD-10-CM: "Pneumonitis due to inhalation of food and vomit"); "J98.19" (ICD-10-CM: "Other pulmonary collapse"); "J96.91" (ICD-10-CM: "Respiratory failure, unspecified with hypoxia"); "J21.8" (ICD-10-CM: "Acute bronchiolitis due to other specified organisms"); "J93.9" (ICD-10-CM: "Pneumothorax, unspecified"); "J98.09" (ICD-10-CM: "Other diseases of bronchus, not elsewhere classified"); "J98.4" (ICD-10-CM: "Other disorders of lung"); "J96.21" (ICD-10-CM: "Acute and chronic respiratory failure with hypoxia"); "J96.22" (ICD-10-CM: "Acute and chronic respiratory failure with hypercapnia"); "J15.9" (ICD-10-CM: "Unspecified bacterial pneumonia"); "J20.9" (ICD-10-CM: "Acute bronchitis, unspecified"); "J45.901" (ICD-10-CM: "Unspecified asthma with (acute) exacerbation"); "J39.8" (ICD-10-CM: "Other specified diseases of upper respiratory tract"); "J96.10" (ICD-10-CM: "Chronic respiratory failure, unspecified whether with hypoxia or hypercapnia"); "J15.1" (ICD-10-CM: "Pneumonia due to Pseudomonas"); "J95.02" (ICD-10-CM: "Infection of tracheostomy stoma"); "J93.0" (ICD-10-CM: "Spontaneous tension pneumothorax"); "J93.83" (ICD-10-CM: "Other pneumothorax"); "J84.9" (ICD-10-CM: "Interstitial pulmonary disease, unspecified"); "J95.821" (ICD-10-CM: "Acute postprocedural respiratory failure"); "J15.4" (ICD-10-CM: "Pneumonia due to other streptococci"); "J38.5" (ICD-10-CM: "Laryngeal spasm"); "J81.0" (ICD-10-CM: "Acute pulmonary edema"); "J12.3" (ICD-10-CM: "Human metapneumovirus pneumonia"); "J45.20" (ICD-10-CM: "Mild intermittent asthma, uncomplicated"); "J14" (ICD-10-CM: "Pneumonia due to Hemophilus influenzae"); "J81.1" (ICD-10-CM: "Chronic pulmonary edema"); "J94.2" (ICD-10-CM: "Hemothorax"); "J99" (ICD-10-CM: "Respiratory disorders in diseases classified elsewhere"); "J10.1" (ICD-10-CM: "Influenza due to other identified influenza virus with other respiratory manifestations"); "J21.9" (ICD-10-CM: "Acute bronchiolitis, unspecified"); "J70.5" (ICD-10-CM: "Respiratory conditions due to smoke inhalation"); "J18.8" (ICD-10-CM: "Other pneumonia, unspecified organism"); "J02.9" (ICD-10-CM: "Acute pharyngitis, unspecified"); "J98.8" (ICD-10-CM: "Other specified respiratory disorders"); "J95.811" (ICD-10-CM: "Postprocedural pneumothorax"); "J96.11" (ICD-10-CM: "Chronic respiratory failure with hypoxia"); "J96.12" (ICD-10-CM: "Chronic respiratory failure with hypercapnia"); "J32.9" (ICD-10-CM: "Chronic sinusitis, unspecified"); "J43.9" (ICD-10-CM: "Emphysema, unspecified"); "J96.20" (ICD-10-CM: "Acute and chronic respiratory failure, unspecified whether with hypoxia or hypercapnia"); "J86.0" (ICD-10-CM: "Pyothorax with fistula"); "J98.2" (ICD-10-CM: "Interstitial emphysema"); "J15.211" (ICD-10-CM: "Pneumonia due to Methicillin susceptible Staphylococcus aureus"); "J15.5" (ICD-10-CM: "Pneumonia due to Escherichia coli"); "J93.82" (ICD-10-CM: "Other air leak"); "J45.31" (ICD-10-CM: "Mild persistent asthma with (acute) exacerbation"); "J35.1" (ICD-10-CM: "Hypertrophy of tonsils"); "J38.4" (ICD-10-CM: "Edema of larynx"); "J96.92" (ICD-10-CM: "Respiratory failure, unspecified with hypercapnia"); "J21.1" (ICD-10-CM: "Acute bronchiolitis due to human metapneumovirus"); "J38.02" (ICD-10-CM: "Paralysis of vocal cords and larynx, bilateral"); "J95.03" (ICD-10-CM: "Malfunction of tracheostomy stoma"); "J38.6" (ICD-10-CM: "Stenosis of larynx"); "J45.40" (ICD-10-CM: "Moderate persistent asthma, uncomplicated"); "J12.9" (ICD-10-CM: "Viral pneumonia, unspecified"); "J02.0" (ICD-10-CM: "Streptococcal pharyngitis"); "J95.1" (ICD-10-CM: "Acute pulmonary insufficiency following thoracic surgery"); "J15.0" (ICD-10-CM: "Pneumonia due to Klebsiella pneumoniae"); "J91.8" (ICD-10-CM: "Pleural effusion in other conditions classified elsewhere"); "J95.850" (ICD-10-CM: "Mechanical complication of respirator"); "J01.00" (ICD-10-CM: "Acute maxillary sinusitis, unspecified"); "J01.20" (ICD-10-CM: "Acute ethmoidal sinusitis, unspecified"); "J15.6" (ICD-10-CM: "Pneumonia due to other Gram-negative bacteria"); "J38.7" (ICD-10-CM: "Other diseases of larynx"); "J45.50" (ICD-10-CM: "Severe persistent asthma, uncomplicated"); "J68.0" (ICD-10-CM: "Bronchitis and pneumonitis due to chemicals, gases, fumes and vapors"); "J30.89" (ICD-10-CM: "Other allergic rhinitis"); "J38.01" (ICD-10-CM: "Paralysis of vocal cords and larynx, unilateral"); "J03.90" (ICD-10-CM: "Acute tonsillitis, unspecified"); "J35.01" (ICD-10-CM: "Chronic tonsillitis"); "J35.02" (ICD-10-CM: "Chronic adenoiditis"); "J35.3" (ICD-10-CM: "Hypertrophy of tonsils with hypertrophy of adenoids"); "J15.212" (ICD-10-CM: "Pneumonia due to Methicillin resistant Staphylococcus aureus"); "J38.00" (ICD-10-CM: "Paralysis of vocal cords and larynx, unspecified"); "J15.20" (ICD-10-CM: "Pneumonia due to staphylococcus, unspecified"); "J98.01" (ICD-10-CM: "Acute bronchospasm"); "J95.01" (ICD-10-CM: "Hemorrhage from tracheostomy stoma"); "J98.6" (ICD-10-CM: "Disorders of diaphragm"); "J35.2" (ICD-10-CM: "Hypertrophy of adenoids"); "J05.10" (ICD-10-CM: "Acute epiglottitis without obstruction"); "J44.9" (ICD-10-CM: "Chronic obstructive pulmonary disease, unspecified"); "J95.851" (ICD-10-CM: "Ventilator associated pneumonia"); "J95.84" (ICD-10-CM: "Transfusion-related acute lung injury (TRALI)"); "J84.01" (ICD-10-CM: "Alveolar proteinosis") |
| Injury, poisoning and certain other consequences of external causes | "909.3" (ICD-9-CM: "Late effect of complications of surgical and medical care"); "996.63" (ICD-9-CM: "Infection and inflammatory reaction due to nervous system device, implant, and graft"); "994.1" (ICD-9-CM: "Drowning and nonfatal submersion"); "989.9" (ICD-9-CM: "Toxic effect of unspecified substance, chiefly nonmedicinal as to source"); "991.6" (ICD-9-CM: "Hypothermia"); "996.68" (ICD-9-CM: "Infection and inflammatory reaction due to peritoneal dialysis catheter"); "879.8" (ICD-9-CM: "Open wound(s) (multiple) of unspecified site(s), without mention of complication"); "800.35" (ICD-9-CM: "Closed fracture of vault of skull with other and unspecified intracranial hemorrhage, with prolonged [more than 24 hours] loss of consciousness, without return to pre-existing conscious level"); "801.2" (ICD-9-CM: "Closed fracture of base of skull with subarachnoid, subdural, and extradural hemorrhage, unspecified state of consciousness"); "852.26" (ICD-9-CM: "Subdural hemorrhage following injury without mention of open intracranial wound, with loss of consciousness of unspecified duration"); "853.06" (ICD-9-CM: "Other and unspecified intracranial hemorrhage following injury without mention of open intracranial wound, with loss of consciousness of unspecified duration"); "862.8" (ICD-9-CM: "Injury to multiple and unspecified intrathoracic organs, without mention of open wound into cavity"); "868" (ICD-9-CM: "Injury to other intra-abdominal organs without mention of open wound into cavity, unspecified intra-abdominal organ"); "922.2" (ICD-9-CM: "Contusion of abdominal wall"); "959.01" (ICD-9-CM: "Head injury, unspecified"); "959.09" (ICD-9-CM: "Injury of face and neck"); "959.8" (ICD-9-CM: "Other specified sites, including multiple injury"); "854" (ICD-9-CM: "Intracranial injury of other and unspecified nature without mention of open intracranial wound, unspecified state of consciousness"); "995.54" (ICD-9-CM: "Child physical abuse"); "854.01" (ICD-9-CM: "Intracranial injury of other and unspecified nature without mention of open intracranial wound, with no loss of consciousness"); "850.4" (ICD-9-CM: "Concussion with prolonged loss of consciousness, without return to pre-existing conscious level (deprecated 2018)"); "873.8" (ICD-9-CM: "Other and unspecified open wound of head without mention of complication"); "929.9" (ICD-9-CM: "Crushing injury of unspecified site"); "829" (ICD-9-CM: "Fracture of unspecified bone, closed"); "864.04" (ICD-9-CM: "Injury to liver without mention of open wound into cavity, laceration, major"); "959.9" (ICD-9-CM: "Unspecified site injury"); "862.9" (ICD-9-CM: "Injury to multiple and unspecified intrathoracic organs, with open wound into cavity"); "890" (ICD-9-CM: "Open wound of hip and thigh, without mention of complication"); "802.38" (ICD-9-CM: "Open fracture of mandible, body, other and unspecified"); "958.3" (ICD-9-CM: "Posttraumatic wound infection not elsewhere classified"); "999.9" (ICD-9-CM: "Other and unspecified complications of medical care, not elsewhere classified"); "800" (ICD-9-CM: "Closed fracture of vault of skull without mention of intracranial injury, unspecified state of consciousness"); "802.8" (ICD-9-CM: "Closed fracture of other facial bones"); "803.6" (ICD-9-CM: "Other open skull fracture with cerebral laceration and contusion, unspecified state of consciousness"); "813.83" (ICD-9-CM: "Closed fracture of unspecified part of radius with ulna"); "853" (ICD-9-CM: "Other and unspecified intracranial hemorrhage following injury without mention of open intracranial wound, unspecified state of consciousness"); "861.21" (ICD-9-CM: "Contusion of lung without mention of open wound into thorax"); "872.8" (ICD-9-CM: "Open wound of ear, part unspecified, without mention of complication"); "873" (ICD-9-CM: "Open wound of scalp, without mention of complication"); "873.41" (ICD-9-CM: "Open wound of cheek, without mention of complication"); "873.63" (ICD-9-CM: "Open wound of tooth (broken) (fractured) (due to trauma), without mention of complication"); "884.1" (ICD-9-CM: "Multiple and unspecified open wound of upper limb, complicated"); "807.09" (ICD-9-CM: "Closed fracture of multiple ribs, unspecified"); "812.41" (ICD-9-CM: "Closed supracondylar fracture of humerus"); "812.43" (ICD-9-CM: "Closed fracture of medial condyle of humerus"); "862" (ICD-9-CM: "Injury to diaphragm, without mention of open wound into cavity"); "864.05" (ICD-9-CM: "Injury to liver without mention of open wound into cavity laceration, unspecified"); "865.09" (ICD-9-CM: "Other injury into spleen without mention of open wound into cavity"); "854.06" (ICD-9-CM: "Intracranial injury of other and unspecified nature without mention of open intracranial wound, with loss of consciousness of unspecified duration"); "996.59" (ICD-9-CM: "Mechanical complication due to other implant and internal device, not elsewhere classified"); "852.03" (ICD-9-CM: "Subarachnoid hemorrhage following injury without mention of open intracranial wound, with moderate [1-24 hours] loss of consciousness"); "854.03" (ICD-9-CM: "Intracranial injury of other and unspecified nature without mention of open intracranial wound, with moderate [1-24 hours] loss of consciousness"); "958.4" (ICD-9-CM: "Traumatic shock"); "821.01" (ICD-9-CM: "Closed fracture of shaft of femur"); "847" (ICD-9-CM: "Sprain of neck"); "852.02" (ICD-9-CM: "Subarachnoid hemorrhage following injury without mention of open intracranial wound, with brief [less than one hour] loss of consciousness"); "897.4" (ICD-9-CM: "Traumatic amputation of leg(s) (complete) (partial), unilateral, level not specified, without mention of complication"); "904.9" (ICD-9-CM: "Injury to blood vessels of unspecified site"); "959.7" (ICD-9-CM: "Knee, leg, ankle, and foot injury"); "E942.0" (ICD-9-CM: "Cardiac rhythm regulators causing adverse effects in therapeutic use"); "879.2" (ICD-9-CM: "Open wound of abdominal wall, anterior, without mention of complication"); "958.92" (ICD-9-CM: "Traumatic compartment syndrome of lower extremity"); "801.03" (ICD-9-CM: "Closed fracture of base of skull without mention of intra cranial injury, with moderate [1-24 hours] loss of consciousness (deprecated 2018)"); "839.01" (ICD-9-CM: "Closed dislocation, first cervical vertebra"); "860.4" (ICD-9-CM: "Traumatic pneumohemothorax without mention of open wound into thorax"); "865.03" (ICD-9-CM: "Injury to spleen without mention of open wound into cavity, laceration extending into parenchyma"); "868.03" (ICD-9-CM: "Injury to other intra-abdominal organs without mention of open wound into cavity, peritoneum"); "868.04" (ICD-9-CM: "Injury to other intra-abdominal organs without mention of open wound into cavity, retroperitoneum"); "873.42" (ICD-9-CM: "Open wound of forehead, without mention of complication"); "873.44" (ICD-9-CM: "Open wound of jaw, without mention of complication"); "933.1" (ICD-9-CM: "Foreign body in larynx"); "800.21" (ICD-9-CM: "Closed fracture of vault of skull with subarachnoid, subdural, and extradural hemorrhage, with no loss of consciousness"); "E911" (ICD-9-CM: "Inhalation and ingestion of food causing obstruction of respiratory tract or suffocation"); "864" (ICD-9-CM: "Injury to liver without mention of open wound into cavity, unspecified injury"); "947.9" (ICD-9-CM: "Burn of internal organs, unspecified site"); "986" (ICD-9-CM: "Toxic effect of carbon monoxide"); "987.7" (ICD-9-CM: "Toxic effect of hydrocyanic acid gas"); "E868.8" (ICD-9-CM: "Accidental poisoning by carbon monoxide from other sources"); "E982.1" (ICD-9-CM: "Poisoning by other carbon monoxide, undetermined whether accidentally or purposely inflicted"); "800.16" (ICD-9-CM: "Closed fracture of vault of skull with cerebral laceration and contusion, with loss of consciousness of unspecified duration"); "803.09" (ICD-9-CM: "Other closed skull fracture without mention of intracranial injury, with concussion, unspecified"); "810" (ICD-9-CM: "Closed fracture of clavicle, unspecified part"); "810.02" (ICD-9-CM: "Closed fracture of shaft of clavicle"); "812" (ICD-9-CM: "Closed fracture of unspecified part of upper end of humerus"); "812.09" (ICD-9-CM: "Other closed fracture of upper end of humerus"); "813.18" (ICD-9-CM: "Open fracture of radius with ulna, upper end (any part)"); "813.23" (ICD-9-CM: "Closed fracture of shaft of radius with ulna"); "813.33" (ICD-9-CM: "Open fracture of shaft of radius with ulna"); "813.44" (ICD-9-CM: "Closed fracture of lower end of radius with ulna"); "813.9" (ICD-9-CM: "Open fracture of unspecified part of forearm"); "821.11" (ICD-9-CM: "Open fracture of shaft of femur"); "821.2" (ICD-9-CM: "Closed fracture of lower end of femur, unspecified part"); "823.2" (ICD-9-CM: "Closed fracture of shaft of tibia alone"); "823.21" (ICD-9-CM: "Closed fracture of shaft of fibula alone"); "824.8" (ICD-9-CM: "Unspecified fracture of ankle, closed"); "864.09" (ICD-9-CM: "Other injury to liver without mention of open wound into cavity"); "850.5" (ICD-9-CM: "Concussion with loss of consciousness of unspecified duration"); "852" (ICD-9-CM: "Subarachnoid hemorrhage following injury without mention of open intracranial wound, unspecified state of consciousness"); "852.05" (ICD-9-CM: "Subarachnoid hemorrhage following injury without mention of open intracranial wound, with prolonged [more than 24 hours] loss of consciousness without return to pre-existing conscious level"); "852.2" (ICD-9-CM: "Subdural hemorrhage following injury without mention of open intracranial wound, unspecified state of consciousness"); "852.25" (ICD-9-CM: "Subdural hemorrhage following injury without mention of open intracranial wound, with prolonged [more than 24 hours] loss of consciousness without return to pre-existing conscious level"); "853.05" (ICD-9-CM: "Other and unspecified intracranial hemorrhage following injury without mention of open intracranial wound, with prolonged [more than 24 hours] loss of consciousness without return to pre-existing conscious level"); "946.3" (ICD-9-CM: "Full-thickness skin loss [third degree NOS] of multiple specified sites"); "948" (ICD-9-CM: "Burn [any degree] involving less than 10 percent of body surface with third degree burn, less than 10 percent or unspecified"); "803.36" (ICD-9-CM: "Other closed skull fracture with other and unspecified intracranial hemorrhage, with loss of consciousness of unspecified duration"); "942.22" (ICD-9-CM: "Blisters, epidermal loss [second degree] of chest wall, excluding breast and nipple"); "944.3" (ICD-9-CM: "Full-thickness skin loss [third degree, not otherwise specified] of hand, unspecified site"); "944.31" (ICD-9-CM: "Full-thickness skin loss [third degree, not otherwise specified] of single digit [finger (nail)] other than thumb"); "944.34" (ICD-9-CM: "Full-thickness skin loss [third degree, not otherwise specified] of two or more digits of hand including thumb"); "944.44" (ICD-9-CM: "Deep necrosis of underlying tissues [deep third degree] without mention of loss of a body part, two or more digits of hand including thumb"); "944.45" (ICD-9-CM: "Deep necrosis of underlying tissues [deep third degree] without mention of loss of a body part, of palm of hand"); "949" (ICD-9-CM: "Burn of unspecified site, unspecified degree"); "801.75" (ICD-9-CM: "Open fracture of base of skull with subarachnoid, subdural, and extradural hemorrhage, with prolonged [more than 24 hours] loss of consciousness, without return to pre-existing conscious level"); "806.2" (ICD-9-CM: "Closed fracture of T1-T6 level with unspecified spinal cord injury"); "862.22" (ICD-9-CM: "Injury to esophagus without mention of open wound into cavity"); "919" (ICD-9-CM: "Abrasion or friction burn of other, multiple, and unspecified sites, without mention of infection"); "872.61" (ICD-9-CM: "Open wound of ear drum, without mention of complication"); "891" (ICD-9-CM: "Open wound of knee, leg [except thigh], and ankle, without mention of complication"); "911" (ICD-9-CM: "Abrasion or friction burn of trunk, without mention of infection"); "912" (ICD-9-CM: "Abrasion or friction burn of shoulder and upper arm, without mention of infection"); "916" (ICD-9-CM: "Abrasion or friction burn of hip, thigh, leg, and ankle, without mention of infection"); "918.1" (ICD-9-CM: "Superficial injury of cornea"); "920" (ICD-9-CM: "Contusion of face, scalp, and neck except eye(s)"); "E860" (ICD-9-CM: "Accidental poisoning by alcohol, not elsewhere classified"); "879.3" (ICD-9-CM: "Open wound of abdominal wall, anterior, complicated"); "879.5" (ICD-9-CM: "Open wound of abdominal wall, lateral, complicated"); "977.9" (ICD-9-CM: "Poisoning by unspecified drug or medicinal substance"); "E858.9" (ICD-9-CM: "Accidental poisoning by unspecified drug"); "E858.8" (ICD-9-CM: "Accidental poisoning by other specified drugs"); "E866.8" (ICD-9-CM: "Accidental poisoning by other specified solid or liquid substances"); "803.06" (ICD-9-CM: "Other closed skull fracture without mention of intracranial injury, with loss of consciousness of unspecified duration"); "801.01" (ICD-9-CM: "Closed fracture of base of skull without mention of intra cranial injury, with no loss of consciousness (deprecated 2018)"); "924.8" (ICD-9-CM: "Contusion of multiple sites, not elsewhere classified"); "928.2" (ICD-9-CM: "Crushing injury of foot"); "807.04" (ICD-9-CM: "Closed fracture of four ribs"); "812.21" (ICD-9-CM: "Closed fracture of shaft of humerus"); "813.22" (ICD-9-CM: "Closed fracture of shaft of ulna (alone)"); "817" (ICD-9-CM: "Multiple closed fractures of hand bones"); "821" (ICD-9-CM: "Closed fracture of unspecified part of femur"); "821.29" (ICD-9-CM: "Other closed fracture of lower end of femur"); "825.25" (ICD-9-CM: "Closed fracture of metatarsal bone(s)"); "827" (ICD-9-CM: "Other, multiple and ill-defined fractures of lower limb, closed"); "839.05" (ICD-9-CM: "Closed dislocation, fifth cervical vertebra"); "853.01" (ICD-9-CM: "Other and unspecified intracranial hemorrhage following injury without mention of open intracranial wound, with no loss of consciousness"); "872.01" (ICD-9-CM: "Open wound of auricle, ear, without mention of complication"); "873.43" (ICD-9-CM: "Open wound of lip, without mention of complication"); "873.64" (ICD-9-CM: "Open wound of tongue and floor of mouth, without mention of complication"); "874.8" (ICD-9-CM: "Open wound of other and unspecified parts of neck, without mention of complication"); "952.05" (ICD-9-CM: "C5-C7 level with unspecified spinal cord injury"); "803" (ICD-9-CM: "Other closed skull fracture without mention of intracranial injury, unspecified state of consciousness"); "869" (ICD-9-CM: "Internal injury to unspecified or ill-defined organs without mention of open wound into cavity"); "813.42" (ICD-9-CM: "Other closed fractures of distal end of radius (alone)"); "910" (ICD-9-CM: "Abrasion or friction burn of face, neck, and scalp except eye, without mention of infection"); "995.55" (ICD-9-CM: "Shaken baby syndrome"); "805.01" (ICD-9-CM: "Closed fracture of first cervical vertebra"); "900.1" (ICD-9-CM: "Injury to internal jugular vein"); "900.89" (ICD-9-CM: "Injury to other specified blood vessels of head and neck"); "854.05" (ICD-9-CM: "Intracranial injury of other and unspecified nature without mention of open intracranial wound, with prolonged [more than 24 hours] loss of consciousness without return to pre-existing conscious level"); "852.21" (ICD-9-CM: "Subdural hemorrhage following injury without mention of open intracranial wound, with no loss of consciousness"); "805.2" (ICD-9-CM: "Closed fracture of dorsal [thoracic] vertebra without mention of spinal cord injury"); "860" (ICD-9-CM: "Traumatic pneumothorax without mention of open wound into thorax"); "860.2" (ICD-9-CM: "Traumatic hemothorax without mention of open wound into thorax"); "922.1" (ICD-9-CM: "Contusion of chest wall"); "874.9" (ICD-9-CM: "Open wound of other and unspecified parts of neck, complicated"); "900.9" (ICD-9-CM: "Injury to unspecified blood vessel of head and neck"); "V54.19" (ICD-9-CM: "Aftercare for healing traumatic fracture of other bone"); "852.09" (ICD-9-CM: "Subarachnoid hemorrhage following injury without mention of open intracranial wound, with concussion, unspecified"); "852.23" (ICD-9-CM: "Subdural hemorrhage following injury without mention of open intracranial wound, with moderate [1-24 hours] loss of consciousness"); "853.09" (ICD-9-CM: "Other and unspecified intracranial hemorrhage following injury without mention of open intracranial wound, with concussion, unspecified"); "851.86" (ICD-9-CM: "Other and unspecified cerebral laceration and contusion, without mention of open intracranial wound, with loss of consciousness of unspecified duration"); "972.6" (ICD-9-CM: "Poisoning by other antihypertensive agents"); "801" (ICD-9-CM: "Closed fracture of base of skull without mention of intra cranial injury, unspecified state of consciousness (deprecated 2018)"); "803.2" (ICD-9-CM: "Other closed skull fracture with subarachnoid, subdural, and extradural hemorrhage, unspecified state of consciousness"); "804.2" (ICD-9-CM: "Closed fractures involving skull or face with other bones with subarachnoid, subdural, and extradural hemorrhage, unspecified state of consciousness"); "923" (ICD-9-CM: "Contusion of shoulder region"); "800.46" (ICD-9-CM: "Closed fracture of vault of skull with intracranial injury of other and unspecified nature, with loss of consciousness of unspecified duration"); "852.31" (ICD-9-CM: "Subdural hemorrhage following injury with open intracranial wound, with no loss of consciousness"); "995.5" (ICD-9-CM: "Child abuse, unspecified"); "959.3" (ICD-9-CM: "Elbow, forearm, and wrist injury"); "861.2" (ICD-9-CM: "Unspecified injury of lung without mention of open wound into thorax"); "996.8" (ICD-9-CM: "Complications of transplanted organ, unspecified"); "801.85" (ICD-9-CM: "Open fracture of base of skull with other and unspecified intracranial hemorrhage, with prolonged [more than 24 hours] loss of consciousness, without return to pre-existing conscious level"); "820.8" (ICD-9-CM: "Closed fracture of unspecified part of neck of femur"); "823.82" (ICD-9-CM: "Closed fracture of unspecified part of fibula with tibia"); "864.01" (ICD-9-CM: "Injury to liver without mention of open wound into cavity, hematoma and contusion"); "864.02" (ICD-9-CM: "Injury to liver without mention of open wound into cavity, laceration, minor"); "865" (ICD-9-CM: "Injury to spleen without mention of open wound into cavity, unspecified injury"); "873.4" (ICD-9-CM: "Open wound of face, unspecified site, without mention of complication"); "873.51" (ICD-9-CM: "Open wound of cheek, complicated"); "873.7" (ICD-9-CM: "Open wound of mouth, unspecified site, complicated"); "959.11" (ICD-9-CM: "Other injury of chest wall"); "800.11" (ICD-9-CM: "Closed fracture of vault of skull with cerebral laceration and contusion, with no loss of consciousness"); "800.31" (ICD-9-CM: "Closed fracture of vault of skull with other and unspecified intracranial hemorrhage, with no loss of consciousness"); "851.41" (ICD-9-CM: "Cerebellar or brain stem contusion without mention of open intracranial wound, with no loss of consciousness"); "924.9" (ICD-9-CM: "Contusion of unspecified site"); "887" (ICD-9-CM: "Traumatic amputation of arm and hand (complete) (partial), unilateral, below elbow, without mention of complication"); "887.1" (ICD-9-CM: "Traumatic amputation of arm and hand (complete) (partial), unilateral, below elbow, complicated"); "887.4" (ICD-9-CM: "Traumatic amputation of arm and hand (complete) (partial), unilateral, level not specified, without mention of complication"); "892.1" (ICD-9-CM: "Open wound of foot except toe(s) alone, complicated"); "896" (ICD-9-CM: "Traumatic amputation of foot (complete) (partial), unilateral, without mention of complication"); "896.1" (ICD-9-CM: "Traumatic amputation of foot (complete) (partial), unilateral, complicated"); "897.1" (ICD-9-CM: "Traumatic amputation of leg(s) (complete) (partial), unilateral, below knee, complicated"); "958.7" (ICD-9-CM: "Traumatic subcutaneous emphysema"); "806.01" (ICD-9-CM: "Closed fracture of C1-C4 level with complete lesion of cord"); "839" (ICD-9-CM: "Closed dislocation, cervical vertebra, unspecified"); "952" (ICD-9-CM: "C1-C4 level with unspecified spinal cord injury"); "959.12" (ICD-9-CM: "Other injury of abdomen"); "998.9" (ICD-9-CM: "Unspecified complication of procedure, not elsewhere classified"); "800.24" (ICD-9-CM: "Closed fracture of vault of skull with subarachnoid, subdural, and extradural hemorrhage, with prolonged [more than 24 hours] loss of consciousness and return to pre-existing conscious level"); "802" (ICD-9-CM: "Closed fracture of nasal bones"); "803.3" (ICD-9-CM: "Other closed skull fracture with other and unspecified intracranial hemorrhage, unspecified state of unconsciousness"); "803.39" (ICD-9-CM: "Other closed skull fracture with other and unspecified intracranial hemorrhage, with concussion, unspecified"); "852.29" (ICD-9-CM: "Subdural hemorrhage following injury without mention of open intracranial wound, with concussion, unspecified"); "800.25" (ICD-9-CM: "Closed fracture of vault of skull with subarachnoid, subdural, and extradural hemorrhage, with prolonged [more than 24 hours] loss of consciousness, without return to pre-existing conscious level"); "800.4" (ICD-9-CM: "Closed fracture of vault of skull with intracranial injury of other and unspecified nature, unspecified state of consciousness"); "998.89" (ICD-9-CM: "Other specified complications of procedures not elsewhere classified"); "800.75" (ICD-9-CM: "Open fracture of vault of skull with subarachnoid, subdural, and extradural hemorrhage, with prolonged [more than 24 hours] loss of consciousness, without return to pre-existing conscious level"); "996.74" (ICD-9-CM: "Other complications due to other vascular device, implant, and graft"); "866.02" (ICD-9-CM: "Injury to kidney without mention of open wound into cavity, laceration"); "934.9" (ICD-9-CM: "Foreign body in respiratory tree, unspecified"); "972" (ICD-9-CM: "Poisoning by cardiac rhythm regulators"); "909.9" (ICD-9-CM: "Late effect of other and unspecified external causes"); "996.1" (ICD-9-CM: "Mechanical complication of other vascular device, implant, and graft"); "T75.1XXA" (ICD-10-CM: "Unspecified effects of drowning and nonfatal submersion, initial encounter"); "T68.XXXA" (ICD-10-CM: "Hypothermia, initial encounter"); "S21.339A" (ICD-10-CM: "Puncture wound without foreign body of unspecified front wall of thorax with penetration into thoracic cavity, initial encounter"); "S41.131A" (ICD-10-CM: "Puncture wound without foreign body of right upper arm, initial encounter"); "T20.15XA" (ICD-10-CM: "Burn of first degree of scalp [any part], initial encounter"); "T20.20XA" (ICD-10-CM: "Burn of second degree of head, face, and neck, unspecified site, initial encounter"); "T23.101A" (ICD-10-CM: "Burn of first degree of right hand, unspecified site, initial encounter"); "T23.102A" (ICD-10-CM: "Burn of first degree of left hand, unspecified site, initial encounter"); "T24.101A" (ICD-10-CM: "Burn of first degree of unspecified site of right lower limb, except ankle and foot, initial encounter"); "T24.102A" (ICD-10-CM: "Burn of first degree of unspecified site of left lower limb, except ankle and foot, initial encounter"); "T31.33" (ICD-10-CM: "Burns involving 30-39% of body surface with 30-39% third degree burns"); "S02.91XA" (ICD-10-CM: "Unspecified fracture of skull, initial encounter for closed fracture"); "S06.1X0A" (ICD-10-CM: "Traumatic cerebral edema without loss of consciousness, initial encounter"); "S06.6X0A" (ICD-10-CM: "Traumatic subarachnoid hemorrhage without loss of consciousness, initial encounter"); "S01.90XA" (ICD-10-CM: "Unspecified open wound of unspecified part of head, initial encounter"); "S00.532A" (ICD-10-CM: "Contusion of oral cavity, initial encounter"); "S06.300A" (ICD-10-CM: "Unspecified focal traumatic brain injury without loss of consciousness, initial encounter"); "S06.5X9A" (ICD-10-CM: "Traumatic subdural hemorrhage with loss of consciousness of unspecified duration, initial encounter"); "S06.6X9A" (ICD-10-CM: "Traumatic subarachnoid hemorrhage with loss of consciousness of unspecified duration, initial encounter"); "S02.119A" (ICD-10-CM: "Unspecified fracture of occiput, initial encounter for closed fracture"); "S09.90XA" (ICD-10-CM: "Unspecified injury of head, initial encounter"); "S82.101A" (ICD-10-CM: "Unspecified fracture of upper end of right tibia, initial encounter for closed fracture"); "S21.132A" (ICD-10-CM: "Puncture wound without foreign body of left front wall of thorax without penetration into thoracic cavity, initial encounter"); "S31.109A" (ICD-10-CM: "Unspecified open wound of abdominal wall, unspecified quadrant without penetration into peritoneal cavity, initial encounter"); "S31.139A" (ICD-10-CM: "Puncture wound of abdominal wall without foreign body, unspecified quadrant without penetration into peritoneal cavity, initial encounter"); "S39.91XA" (ICD-10-CM: "Unspecified injury of abdomen, initial encounter"); "S05.20XA" (ICD-10-CM: "Ocular laceration and rupture with prolapse or loss of intraocular tissue, unspecified eye, initial encounter"); "S05.21XA" (ICD-10-CM: "Ocular laceration and rupture with prolapse or loss of intraocular tissue, right eye, initial encounter"); "S06.369A" (ICD-10-CM: "Traumatic hemorrhage of cerebrum, unspecified, with loss of consciousness of unspecified duration, initial encounter"); "T14.90XA" (ICD-10-CM: "Injury, unspecified, initial encounter"); "S41.002A" (ICD-10-CM: "Unspecified open wound of left shoulder, initial encounter"); "S21.102A" (ICD-10-CM: "Unspecified open wound of left front wall of thorax without penetration into thoracic cavity, initial encounter"); "S27.1XXA" (ICD-10-CM: "Traumatic hemothorax, initial encounter"); "S31.103A" (ICD-10-CM: "Unspecified open wound of abdominal wall, right lower quadrant without penetration into peritoneal cavity, initial encounter"); "S41.102A" (ICD-10-CM: "Unspecified open wound of left upper arm, initial encounter"); "T79.4XXA" (ICD-10-CM: "Traumatic shock, initial encounter"); "S06.5X0A" (ICD-10-CM: "Traumatic subdural hemorrhage without loss of consciousness, initial encounter"); "S22.43XA" (ICD-10-CM: "Multiple fractures of ribs, bilateral, initial encounter for closed fracture"); "S22.49XA" (ICD-10-CM: "Multiple fractures of ribs, unspecified side, initial encounter for closed fracture"); "S40.872A" (ICD-10-CM: "Other superficial bite of left upper arm, initial encounter"); "S50.871A" (ICD-10-CM: "Other superficial bite of right forearm, initial encounter"); "T74.12XA" (ICD-10-CM: "Child physical abuse, confirmed, initial encounter"); "T85.611A" (ICD-10-CM: "Breakdown (mechanical) of intraperitoneal dialysis catheter, initial encounter"); "T85.71XA" (ICD-10-CM: "Infection and inflammatory reaction due to peritoneal dialysis catheter, initial encounter"); "S01.83XA" (ICD-10-CM: "Puncture wound without foreign body of other part of head, initial encounter"); "S01.93XA" (ICD-10-CM: "Puncture wound without foreign body of unspecified part of head, initial encounter"); "S02.0XXA" (ICD-10-CM: "Fracture of vault of skull, initial encounter for closed fracture"); "S02.0XXB" (ICD-10-CM: "Fracture of vault of skull, initial encounter for open fracture"); "S05.41XA" (ICD-10-CM: "Penetrating wound of orbit with or without foreign body, right eye, initial encounter"); "S06.2X0A" (ICD-10-CM: "Diffuse traumatic brain injury without loss of consciousness, initial encounter"); "S06.360A" (ICD-10-CM: "Traumatic hemorrhage of cerebrum, unspecified, without loss of consciousness, initial encounter"); "S06.4X0A" (ICD-10-CM: "Epidural hemorrhage without loss of consciousness, initial encounter"); "S29.9XXA" (ICD-10-CM: "Unspecified injury of thorax, initial encounter"); "S19.9XXA" (ICD-10-CM: "Unspecified injury of neck, initial encounter"); "T45.525A" (ICD-10-CM: "Adverse effect of antithrombotic drugs, initial encounter"); "S59.202D" (ICD-10-CM: "Unspecified physeal fracture of lower end of radius, left arm, subsequent encounter for fracture with routine healing"); "S82.192A" (ICD-10-CM: "Other fracture of upper end of left tibia, initial encounter for closed fracture"); "T76.12XA" (ICD-10-CM: "Child physical abuse, suspected, initial encounter"); "S21.139A" (ICD-10-CM: "Puncture wound without foreign body of unspecified front wall of thorax without penetration into thoracic cavity, initial encounter"); "S21.332A" (ICD-10-CM: "Puncture wound without foreign body of left front wall of thorax with penetration into thoracic cavity, initial encounter"); "S26.09XA" (ICD-10-CM: "Other injury of heart with hemopericardium, initial encounter"); "S51.832A" (ICD-10-CM: "Puncture wound without foreign body of left forearm, initial encounter"); "S06.9X9A" (ICD-10-CM: "Unspecified intracranial injury with loss of consciousness of unspecified duration, initial encounter"); "S02.19XA" (ICD-10-CM: "Other fracture of base of skull, initial encounter for closed fracture"); "S02.2XXA" (ICD-10-CM: "Fracture of nasal bones, initial encounter for closed fracture"); "S02.609A" (ICD-10-CM: "Fracture of mandible, unspecified, initial encounter for closed fracture"); "S12.101A" (ICD-10-CM: "Unspecified nondisplaced fracture of second cervical vertebra, initial encounter for closed fracture"); "S22.41XA" (ICD-10-CM: "Multiple fractures of ribs, right side, initial encounter for closed fracture"); "S27.322A" (ICD-10-CM: "Contusion of lung, bilateral, initial encounter"); "S27.329A" (ICD-10-CM: "Contusion of lung, unspecified, initial encounter"); "S36.113A" (ICD-10-CM: "Laceration of liver, unspecified degree, initial encounter"); "S36.116A" (ICD-10-CM: "Major laceration of liver, initial encounter"); "S39.92XA" (ICD-10-CM: "Unspecified injury of lower back, initial encounter"); "S39.93XA" (ICD-10-CM: "Unspecified injury of pelvis, initial encounter"); "S42.001A" (ICD-10-CM: "Fracture of unspecified part of right clavicle, initial encounter for closed fracture"); "S42.031A" (ICD-10-CM: "Displaced fracture of lateral end of right clavicle, initial encounter for closed fracture"); "S49.91XA" (ICD-10-CM: "Unspecified injury of right shoulder and upper arm, initial encounter"); "S49.92XA" (ICD-10-CM: "Unspecified injury of left shoulder and upper arm, initial encounter"); "S59.911A" (ICD-10-CM: "Unspecified injury of right forearm, initial encounter"); "S59.912A" (ICD-10-CM: "Unspecified injury of left forearm, initial encounter"); "S70.12XA" (ICD-10-CM: "Contusion of left thigh, initial encounter"); "S72.322A" (ICD-10-CM: "Displaced transverse fracture of shaft of left femur, initial encounter for closed fracture"); "S72.492A" (ICD-10-CM: "Other fracture of lower end of left femur, initial encounter for closed fracture"); "S79.102A" (ICD-10-CM: "Unspecified physeal fracture of lower end of left femur, initial encounter for closed fracture"); "S82.201A" (ICD-10-CM: "Unspecified fracture of shaft of right tibia, initial encounter for closed fracture"); "S82.202A" (ICD-10-CM: "Unspecified fracture of shaft of left tibia, initial encounter for closed fracture"); "S82.221A" (ICD-10-CM: "Displaced transverse fracture of shaft of right tibia, initial encounter for closed fracture"); "S82.224A" (ICD-10-CM: "Nondisplaced transverse fracture of shaft of right tibia, initial encounter for closed fracture"); "S82.312A" (ICD-10-CM: "Torus fracture of lower end of left tibia, initial encounter for closed fracture"); "S82.401A" (ICD-10-CM: "Unspecified fracture of shaft of right fibula, initial encounter for closed fracture"); "S82.402A" (ICD-10-CM: "Unspecified fracture of shaft of left fibula, initial encounter for closed fracture"); "T07.XXXA" (ICD-10-CM: "Unspecified multiple injuries, initial encounter"); "S00.83XA" (ICD-10-CM: "Contusion of other part of head, initial encounter"); "S21.131A" (ICD-10-CM: "Puncture wound without foreign body of right front wall of thorax without penetration into thoracic cavity, initial encounter"); "S71.132A" (ICD-10-CM: "Puncture wound without foreign body, left thigh, initial encounter"); "T85.890A" (ICD-10-CM: "Other specified complication of nervous system prosthetic devices, implants and grafts, initial encounter"); "S02.69XB" (ICD-10-CM: "Fracture of mandible of other specified site, initial encounter for open fracture"); "S05.02XA" (ICD-10-CM: "Injury of conjunctiva and corneal abrasion without foreign body, left eye, initial encounter"); "S11.23XA" (ICD-10-CM: "Puncture wound without foreign body of pharynx and cervical esophagus, initial encounter") |
